# A coach-supported, digital parenting programme for parents of adolescents at risk of suicide: pilot trial of acceptability, feasibility, validity and short-term effects

**DOI:** 10.1192/bjo.2026.11046

**Published:** 2026-05-19

**Authors:** Alice Cao, Glenn A. Melvin, Mairead Cardamone-Breen, Chloe Salvaris, Patrick Olivier, Ling Wu, Joshua Seguin, Jue Xie, Dhruv Basur, Andrew Thompson, Steven Leicester, Penelope Hasking, Anthony F. Jorm, Marie B. H. Yap

**Affiliations:** School of Psychological Sciences, Turner Institute for Brain and Mental Health, Monash University, Australia; School of Psychology, Centre for Social and Early Emotional Development, Deakin University, Australia; Action Lab, Faculty of Information Technology, Monash University, Australia; Centre for Youth Mental Health, Orygen, Australia; headspace National Youth Mental Health Foundation, Melbourne, Australia; School of Population Health, Curtin enAble Institute, Curtin University, Australia; Melbourne School of Population and Global Health, University of Melbourne, Australia

**Keywords:** Youth, caregiver, online, suicide prevention, clinician

## Abstract

**Background:**

Parents of adolescents facing suicidality play a crucial protective role, but often feel overwhelmed. The Partners in Parenting Plus – Suicide Prevention (PiP-SP+) programme is a co-designed, coach-supported, online parenting programme aimed to empower parents within their carer role to manage their adolescent’s suicide risk.

**Aims:**

To evaluate PiP-SP+’s acceptability, feasibility, validity and short-term effects.

**Method:**

Fifteen parents of adolescents aged 12–18 years, concerned about their adolescent’s suicidality, participated in an open-label, non-randomised uncontrolled trial. Parents (*n* = 11) completed semi-structured interviews, exploring the programme’s acceptability, feasibility and validity. Thirteen parents completed quantitative assessments of parental self-efficacy to respond to adolescent suicidality and non-suicidal self-injury, protective parenting behaviours, carer burden, parental distress, mental health support quality, family functioning, and adolescent anxiety and depressive symptoms at baseline and 120 days post baseline. Finally, nine adolescents of the participating parents self-reported anxiety symptoms, depressive symptoms and perceptions of parental support at baseline and 120 days post baseline.

**Results:**

PiP-SP+ was an acceptable, feasible and valid intervention for parents. Significant baseline-to-post-intervention improvements were observed in parents’ self-efficacy to respond to adolescent suicidality and non-suicidal self-injury, protective parenting behaviours, carer burden, parental distress and mental health support quality. No significant differences were reported in family functioning. Adolescents perceived increased parental support; both parents and adolescents reported reductions in adolescent anxiety symptoms. Although parents reported a significant decrease in adolescent depressive symptoms, adolescents did not.

**Conclusions:**

Findings support the value of undertaking an appropriately powered, randomised controlled trial to confirm these pilot findings.

Anxiety disorders and depression (internalising disorders) are the most prevalent mental disorders in adolescence (aged 12–18 years),^1^ and concerningly, adolescents with internalising disorders are 1.54 times more likely to attempt suicide.^2^ Yet, an estimated 50% of young people aged 12–26 years with suicidal thoughts and behaviours (suicidality) do not seek professional help.^3^ Therefore, the involvement of supports, such as parents, is considered essential for effective adolescent suicide prevention.^4,5^ Parents play a pivotal practical role in suicide prevention, including limiting access to lethal means, providing emotional support and monitoring risk.^6^ Further, parents are the most common source of support that adolescents report turning to if they encounter mental health difficulties.^7^ However, parents of adolescents experiencing suicidality often feel uncertain about how to safeguard their adolescent’s well-being, and lack self-efficacy (confidence) in providing emotional support.^8^ There are very few evidence-based resources for parents of adolescents experiencing suicidality, and most existing interventions are face to face or do not offer personalised support by a mental health professional.^9^ Face-to-face interventions present logistical barriers for many parents (e.g. scheduling conflicts, childcare/transportation arrangements).^10^ Increasingly, research demonstrates parents’ preference for information about parenting and mental health to be delivered digitally,^10^ and the effectiveness of technology-based parenting interventions to promote children’s psychological outcomes.^11^ However, parents of adolescents experiencing suicidality desire tailored emotional, practical and relationship support from mental health professionals,^12^ thus a purely self-directed, digital approach may not respond to the nuanced complexities of the parent–adolescent relationship and the adolescent’s acute clinical risk.^12^ Given these challenges and needs, a coach-supported digital parenting programme appears a promising candidate solution, by offering flexible, tailored support that parents can access without the barriers of traditional face-to-face interventions. To our knowledge, no coach-supported digital parenting programme currently exists; therefore, the Partners in Parenting Plus – Suicide Prevention (PiP-SP+) programme was developed. PiP-SP+ is an adaptation of the Partners in Parenting Plus programme (PiP+), previously known as the Therapist-assisted Online Parenting Strategies programme.^13^ PiP+ is a manualised, coach-supported online parenting programme designed to improve parent-related risk and protective factors for parents of adolescents (aged 12–18 years) experiencing clinical levels of anxiety or depression.^13^ PiP+ constitutes the highest level of the multi-level Partners in Parenting (PiP) programme,^14^ where online modules are complemented by video-conferenced coaching sessions with a trained mental health professional to provide more intensive support for parents of adolescents with clinical levels of anxiety and depression.^13^ Seventy-one parents (94.4% mothers) and their adolescents (73.2% female) aged 12–18 (mean 15.02, s.d. 1.56) years participated in the single-arm, double-baseline clinical trial.^15^ PiP+ was found to be acceptable, feasible and associated with improvements in parental self-efficacy, family functioning and parenting behaviours protective against adolescent anxiety and depression.^15^ Given the co-occurrence of adolescent suicidality and internalising disorders, PiP+ appeared ideally situated for adaptation. The resulting PiP-SP+ intervention is described in detail in the ‘PiP-SP+ intervention’ section.

## Current pilot study

The Medical Research Council provides a step-wise framework for developing and evaluating complex interventions,^16,17^ where the preliminary efficacy of an intervention is established via acceptability, feasibility, validity and short-term effects before conducting a large-scale, randomised controlled trial.^17^ The current study presents the findings of this crucial first step in the evaluation of PiP-SP+, using a mixed-methods evaluation of a pilot trial, to inform future programme development and evaluation.^18^

## Study aims and hypotheses

The study aimed to evaluate the acceptability, feasibility, validity and short-term effects of PiP-SP+ among parents of adolescents concerned about suicide risk in the context of anxiety or depressive difficulties in an open-label, uncontrolled pilot trial. We hypothesised the following:

- PiP-SP+ will be an acceptable, feasible and valid intervention for parents.
- From baseline to post-intervention, parents will report improvements in parenting self-efficacy to respond to adolescent suicidality and non-suicidal self-injury, protective parenting behaviours against adolescent anxiety and depression, family functioning, quality of mental health support provided and carer burden, as well as reductions in anxiety and depression symptoms in their adolescent.
- From baseline to post-intervention, adolescents will report reductions in anxiety and depression symptoms, as well as improvements in their perception of parental emotional support if experiencing suicidal ideation or self-injuring.

## Method

### Trial design and ethical approval

The trial protocol was prospectively registered on the Australian New Zealand Clinical Trials Registry (ACTRN12623000266662) on 13 March 2023, and followed CONSORT reporting recommendations (Supplementary Material A).^19^ The authors assert that all procedures contributing to this work comply with the ethical standards of the relevant national and institutional committees on human experimentation and with the Helsinki Declaration of 1975, as revised in 2013. All procedures involving human participants were approved by Monash University Human Research Ethics Committee (identifier #36057). Following trial commencement, some outcome measures were removed and the sample size was reduced because of research team capacity. However, these changes occurred before any participant commencing the intervention (see Supplementary Material B for further detail).

### Recruitment and inclusion criteria

Participants were recruited across Australia, between March to August 2023, via emailing parents who had expressed interest in a similar trial (identifier ACTRN12622001365752), and provided consent to be contacted about future research opportunities. Further, participants were recruited through professional networks and online social media groups. Parent eligibility criteria were (a) parent of an adolescent (aged 12–18 years), (b) concerned about their adolescent’s suicidal thoughts and/or behaviours, (c) live in Australia, (d) can communicate in English and (e) have regular access to internet, telephone and email. Adolescents of participating parents were required to be engaged with a mental health professional, as PiP-SP+ is not intended as a treatment for the adolescent’s mental health problems or suicidality. Adolescents did not need to be diagnosed with an anxiety disorder or depression.

### Participants

Participants included 15 parents of adolescents aged 13–18 years. The sample size of 15 exceeded that recommended for pilot studies (*N* = 10^20^), and recruitment stopped once the target sample size was surpassed. Nine adolescents participated in pre- and post-intervention assessments. Tables 1 and 2 show parent- and adolescent-reported sample characteristics at baseline.

**Table 1:** Parent-reported sample characteristics for themselves and their adolescent at baseline

Parent characteristic, descriptor (*n* = 15)	
Gender, *n* (%)	
Woman	14 (93.33)
Man	1 (6.67)
Relationship to adolescent, *n* (%)	
Mother	14 (93.33)
Father	1 (6.67)
Country/region of origin, *n* (%)	
Australia/New Zealand	11 (73.33)
Western Europe	2 (13.33)
Other	2 (13.33)
Education (highest qualification), *n* (%)	
Postgraduate degree (Master’s or doctorate)	4 (26.67)
Undergraduate degree	3 (20.00)
Diploma/advanced diploma	4 (26.67)
TAFE/technical course^a^	1 (6.67)
Secondary school	3 (20.00)
Relationship status, *n* (%)	
Married or *de facto*	13 (86.67)
Separated or divorced	2 (13.33)
Government concession card, *n* (%)^b^	
No	14 (93.33)
Yes, for both parent and adolescent	1 (6.67)

Adolescent characteristics by parent-report, descriptor (*n* = 15)	
Gender, *n* (%)	
Girl	13 (86.67)
Boy	2 (13.33)
Age in years, mean (s.d.)	15.88 (1.24)
Adolescent’s total anxiety and depressive symptoms by parent-report (RCADS-25-P total score^c^), mean (s.d.)	33.07 (8.55)
Adolescent’s anxiety symptoms by parent-report (RCADS-25-P anxiety subscale^c^), mean (s.d.)	15.47 (5.76)
Adolescent’s depressive symptoms by parent-report (RCADS-25-P depressive subscale^c^), mean (s.d.)	17.60 (3.74)
Adolescent’s symptoms elevated above clinical threshold by parent-report (RCADS-25-P^c^), *n* (%)	
Total anxiety and depression	13 (86.67)
Total anxiety	9 (60.00)
Total depression	15 (100)
Primary treating mental health professional for adolescent, *n* (%)	
Psychologist	10 (66.67)
Psychiatrist	2 (13.33)
Counsellor	1 (6.67)
General practitioner	1 (6.67)
Occupational therapist	1 (6.67)

a. Technical and Further Education (TAFE) institutes offer vocational and technical education programmes in Australia.

b. Government concession cards are issued by the Australian Government based on income and financial circumstances, and are used in this study as a proxy for low socioeconomic position.

c. Revised Child Anxiety and Depression Scale 25-item – Parent Version (RCADS-25-P) symptom elevation above clinical threshold: T-score ≥70.^21,22^

**Table 2:** Adolescent self-reported characteristics at baseline

Adolescent characteristics (*n* = 9) from self-report at baseline, descriptor	
Gender, *n* (%)	
Girl/woman	7 (77.78)
Boy/man	2 (22.22)
Age, years, mean (s.d.)	15.70 (1.28)
Adolescent’s anxiety and depressive symptoms by self-report (RCADS-25 total score^a^), mean (s.d.)	40.00 (9.85)
Adolescent’s anxiety symptoms by self-report (RCADS-25 anxiety subscale^a^), mean (s.d.)	21.33 (5.70)
Adolescent’s depressive symptoms by self-report (RCADS-25 depressive subscale^a^), mean (s.d.)	18.67 (4.74)
Adolescent’s symptoms elevated above clinical threshold by self-report (RCADS-25^a^), *n* (%)	
Total anxiety and depression	7 (77.78)
Total anxiety	3 (42.86)
Total depression	7 (77.78)

a. Revised Child Anxiety and Depression Scale 25-item version (RCADS-25) symptom elevation above clinical threshold: T-score ≥70.^21,23^

### Procedures

**Fig. 1:**
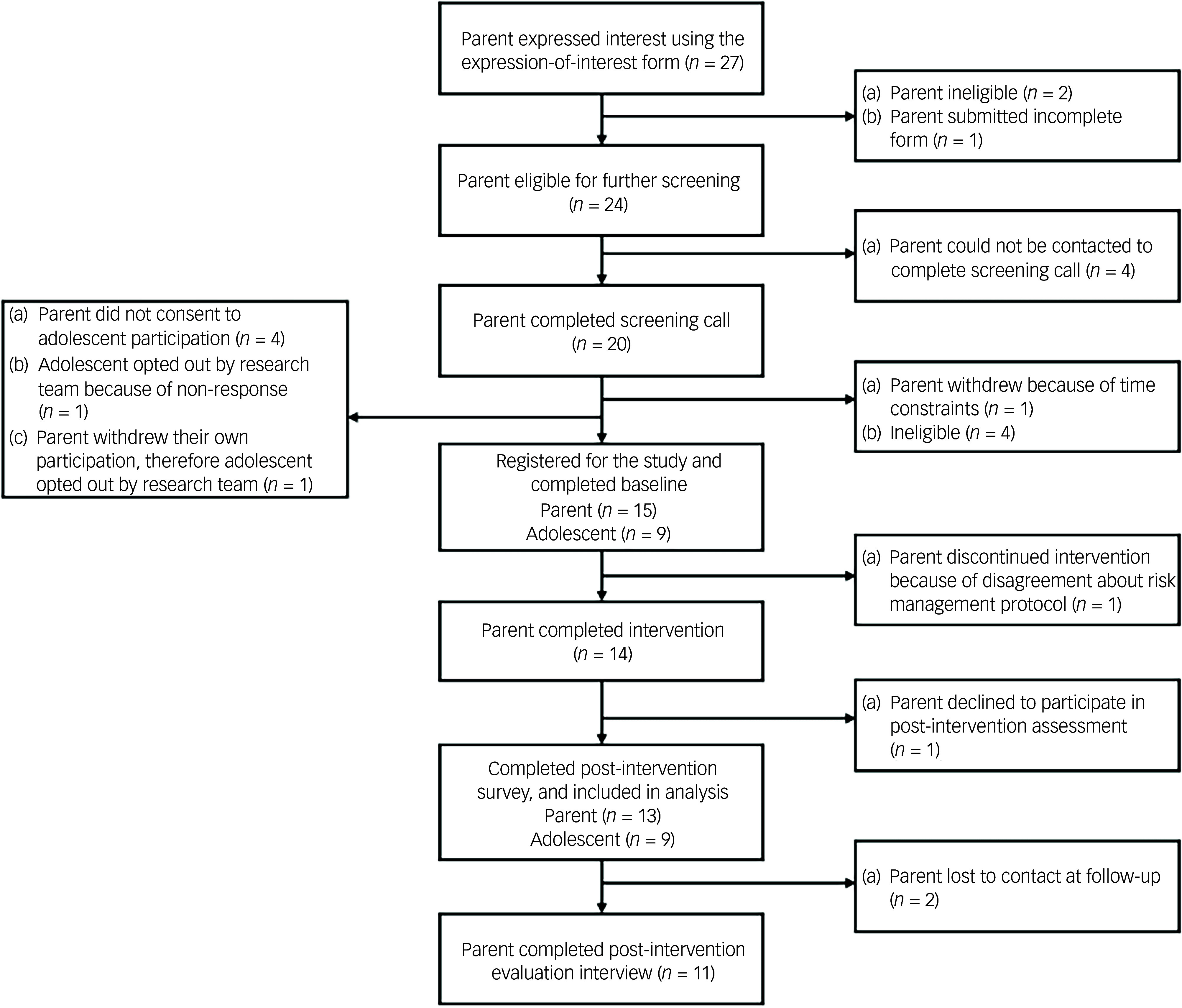
PiP-SP + procedure and participant flow diagram.

Overarching PiP-SP+ procedures and participant flow is presented in Fig. 1.

Interested parents completed an online expression-of-interest form, which was followed by a screening telephone call with a member of the research team (registered or provisional psychologists) to confirm eligibility and discuss the potential involvement of their adolescent in research assessments. To ensure that adolescents were not pressured to participate, the research team (a) explained to parents at the screening call that parental eligibility was not contingent on their adolescent’s participation, and that their adolescent’s participation was optional; and (b) withdrew adolescent participation if after two instances of follow-up the adolescent did not reply to the survey package. Eligible parents reviewed the participant explanatory statement, provided online informed consent (including for their adolescent, if applicable) and provided demographic data about themselves and their adolescent via an electronic data capturing tool, REDCap version 13.4 for macOS (Vanderbilt University, Tennessee, USA, hosted by Monash University, Victoria, Australia; https://redcap.helix.monash.edu/).^24,25^ Parents also received the adolescent explanatory statement and indicated whether they had discussed the study with their adolescent and if their adolescent consented to participate. Participating adolescents were emailed a link to the baseline assessment and received an e-gift voucher (AUD$ 20) upon completion. Adolescents aged 18 years were sent the adolescent explanatory statement and an informed consent form to complete themselves via REDCap. Parents completed their baseline assessment on the trial website and received a tailored feedback report on areas of parenting strengths and areas for development, based on their responses on the Parenting to Reduce Adolescent Depression and Anxiety Scale (PRADAS).^26^ Parents then commenced the PiP-SP+ intervention with a designated coach.

At 120 days post baseline, parents and adolescents were invited to complete the post-intervention assessments via the dedicated trial website (parents) or via REDCap (adolescent) each receiving reimbursement (AUD$ 20).^24,25^ A 120-day post-baseline interval was set as this was the amount of time we anticipated that parents might take to complete the PiP-SP+ intervention, including a buffer (e.g. reschedules). Given that the programme was individually tailored to each parent, the length of the programme varied. As such, parents who completed the intervention close to the 120-day mark completed their survey soon after finishing the PiP-SP+ programme, whereas those who completed the PiP-SP+ intervention earlier may have had several weeks before completing the post-intervention survey.

Additionally, parents were invited to participate in a 45 min semi-structured interview conducted via Zoom by a member of the research team who was not the parent’s coach, for which they received another e-gift voucher reimbursement (AUD$ 20). The interview was completed in a one-on-one format via videoconferencing, with audio and video recording, and securely stored on the university server.

### PiP-SP+ intervention

PiP-SP+ is an adapted version of the PiP+ programme,^13^ with the addition of suicide prevention specific content. PiP-SP+ was developed through phases adhering to the framework for design innovation,^27^ including (a) a review of existing evidence-based best practice suicide prevention strategies from credible sources (e.g. Mental Health First Aid International^28^); (b) interviews conducted with parents, young people and experts in youth mental health/suicide prevention to understand the support needs of parents with adolescents experiencing suicidality (outlined elsewhere^12^); and (c) co-design workshops with stakeholders to develop specific enhancements (outlined elsewhere^29^). Parents were encouraged to complete modules and coaching sessions weekly, with a maximum of 14 weeks to complete the six to nine coaching sessions and associated modules.

#### Online modules and coaching sessions

The PiP-SP+ intervention consisted of up to 13 self-directed, weekly, online modules, including the original 10 PiP modules covering domains associated with adolescent internalising disorders and 3 new modules specific to suicide risk and non-suicidal self-injury (module content is outlined in Supplementary Material C). Alongside the modules, parents had up to nine coaching sessions with their dedicated PiP+ coach (see below section). The programme constituted five ‘core’ required online modules and six coaching sessions, with four additional optional, recommended topics. Core topics were chosen by senior investigators (M.B.H.Y., G.A.M. and A.F.J.) based on their clinical and empirical relevance to adolescent suicide prevention in the context of anxiety and/or depression (outlined in Supplementary Material C).^13^ Parents could select optional modules they would like to complete in addition to the core topics. Modules took approximately 20–35 min each and included educational materials, illustrations, audio clips, videos, vignettes, activities, goal-setting exercises and an end-of-module quiz.

Coaching sessions were delivered by a registered or provisionally -registered psychologist. Parents were required to complete the associated module before attending the coaching session. Each session was approximately 60 min. Coaching sessions were manualised, with each following a similar structure and aiming to facilitate tailoring and support the parent in implementing the parenting recommendations in the modules. Parents were encouraged to complete modules and coaching sessions weekly, with a maximum of 14 weeks to complete the six to nine coaching sessions and associated modules.

Coaching fidelity was supported through training, regular (weekly) group supervision, standardised operating procedures and coaching manuals that provided session agendas and exemplar scripts (coaching content outlined in Supplementary Material C). Supervision was provided by G.A.M., a registered clinical psychologist with extensive clinical expertise in adolescent suicide prevention, and M.B.H.Y., a psychologist and founder and lead researcher of the PiP programme.

#### PiP-SP+ action plan

Parents were provided with a collaborative coach–parent resource housed on Google Slides, called the ‘PiP-SP+ Action Plan’. This resource was used alongside the PiP-SP+ modules and coaching sessions to guide reflection, record learning and summarise progress, with the aim of developing parents’ confidence in responding to their adolescent’s suicidality and providing emotional support. The content of the PiP-SP+ Action Plan was informed by co-design workshops outlined elsewhere;^29^ sample content of the PiP-SP+ Action Plan and its alignment with module material and coaching sessions is provided in Supplementary Material D.

### Measures

Example items and response scales for each quantitative measure are provided in Supplementary Material E.

#### Acceptability, including validity and feasibility

Acceptability was assessed quantitatively and qualitatively at post intervention (120 days post baseline). The eight-item Client Satisfaction Questionnaire (CSQ-8)^30^ was used to quantitatively assess parental satisfaction with the PiP-SP+ programme, with higher scores indicating greater satisfaction. The total scale score ranges from 8 to 32. Cronbach’s *α* (internal consistency) was 0.75. The acceptability of PiP-SP+ was assessed by semi-structured interviews with parents to supplement quantitative outcomes. The interview schedule (Supplementary Material F) was adapted from the Theoretical Framework of Acceptability (TFA)^31^ questionnaire, which is a theory-informed approach to explore the acceptability of a healthcare intervention.

According to the TFA, acceptability includes domains of validity (i.e. perceived effectiveness and intervention coherence) and feasibility (i.e. burden, opportunity costs, intervention coherence and confidence in completing the programme).^31,32^ Therefore, the validity and feasibility of the intervention were explored through the interviews.^32^ Qualitative measures of feasibility were complemented by quantitative measurement of completion and adherence rates. Programme completion was calculated separately for online modules and coaching sessions; for example, 100% × (number of selected modules completed) / (total number of selected modules). Calculations only considered topics that included a module and corresponding coaching session. Programme adherence was defined as the percentage of parents who completed the core programme (the five required online modules and six coaching sessions) out of the overall sample: 100% × [(number of participants who completed the required modules and coaching sessions) / (total number of participants who received the intervention)].

#### Baseline-to-post-intervention outcomes by parent self-report

Parental self-efficacy to respond to adolescent suicidality was measured by parent self-report of ten items adapted from a validated scale assessing parent’s self-efficacy in their ability to prevent or assist their child experiencing suicidality,^8^ with higher scores indicating greater self-efficacy (total scale score ranges from 0 to 100; see Supplementary Material G for adapted scale). Cronbach’s *α* was 0.93 for baseline and 0.93 for post intervention.

Parental self-efficacy to respond to adolescent non-suicidal self-injury was measured by a five-item scale developed for the trial, with higher scores indicating greater self-efficacy (see Supplementary Material H; total scale score ranges from 0 to 50). Cronbach’s *α* was 0.82 for baseline and 0.83 for post intervention.

Parenting behaviours protective against adolescent anxiety and depression was measured by the PRADAS,^26^ a 73-item criterion-referenced scale, with higher scores indicating greater concordance with evidence-based parenting guidelines for the prevention of adolescent depression and anxiety disorders.^33^ The total scale score ranges from 0 to 73. Examples of parenting behaviours include establishing and maintaining parent–adolescent relationships, encouraging supportive friendships and establishing family rules.^33^ Scale reliability, as indicated by agreement coefficients,^34^ was 0.96 for baseline and 0.93 for post-intervention.

Parental self-efficacy to engage in protective parenting behaviours against anxiety and depression was measured by the Parental Self-Efficacy Scale, consisting of eight items.^35^ Higher scores indicate greater parental self-efficacy (total scale score ranges from 8 to 32). Cronbach’s *α* was 0.84 for baseline and 0.82 for post intervention.

Quality of mental health support intended to be provided was measured via 18 items, using an adapted version of the Mental Health Support Scale – Intended^36^ (see Supplementary Material I), where higher total scores indicate higher general knowledge and skills of parents in recognising and responding to mental health problems (total scale scores ranges from 18 to 90). Cronbach’s *α* was 0.63 for baseline and 0.80 for post intervention.

Carer burden was assessed by the 19-item Burden Assessment Scale,^37^ with higher scores indicating greater burden (total scale scores ranges from 19 to 76). Cronbach’s *α* was 0.81 for baseline and 0.75 for post intervention.

Family functioning was assessed by the general functioning subscale of the McMaster Family Assessment Device,^38^ where higher scores indicate greater impairment in family functioning (total scale scores ranges from 12 to 48). Cronbach’s *α* was 0.73 for baseline and 0.85 for post intervention.

Parent psychological distress was self-reported with the Kessler-6 scale,^39^ with higher scores indicating greater distress (total scale scores range from 6 to 30). Cronbach’s *α* was 0.81 for baseline and 0.92 for post intervention.

Parent-report of their adolescent’s anxiety and depressive symptoms was measured by the Revised Child Anxiety and Depression Scale 25-item – Parent Version^22^ anxiety subscale and depressive subscale, respectively, with higher scores indicating greater symptoms (anxiety total subscale score ranges from 0 to 45, and depressive total subscale score ranges from 0 to 30). Cronbach’s *α* of the anxiety subscale was 0.78 for baseline and 0.79 for post intervention, Cronbach’s *α* of the depressive subscale was 0.79 for baseline and 0.80 for post intervention.

#### Baseline-to-post-intervention outcomes by adolescent self-report

Perceived parental emotional support if the adolescent was experiencing suicidal ideation or self-injuring *w*as measured by a four-item scale developed for the trial, with higher scores indicating greater perceived parental emotional support (total scale score ranges from 0 to 16; see Supplementary Material J). Cronbach’s *α* was 0.82 for baseline and 0.67 for post intervention.

Adolescent self-reported anxiety and depressive symptoms were measured by the Revised Child Anxiety and Depression Scale – Short Version^23^ anxiety subscale and depressive subscale, respectively, with higher scores indicating greater symptoms (anxiety total subscale score ranges from 0 to 45, and depressive total subscale score ranges from 0 to 30). Cronbach’s *α* of the anxiety subscale was 0.67 for baseline and 0.79 for post intervention. Cronbach’s *α* of the depressive subscale was 0.77 for baseline and 0.65 for post intervention.

### Data analysis

Interview data were first transcribed and then analysed through the process of reflexive thematic analysis, adhering to the six phases outlined by Braun and Clarke.^40^ Qualitative data was transcribed by trained members of the research team proficient in transcription techniques and protocols. A.C. undertook the complete coding of transcripts. Themes were identified deductively against the TFA questionnaire,^31^ as the TFA provided predefined constructs that constitute acceptability (e.g. perceived effectiveness and intervention coherence); additional subthemes were identified inductively. Therefore, the validity and feasibility of the intervention were also explored through the interviews. Candidate subthemes were recursively developed and discussed within the research team until the subthemes were conceptualised.

For all quantitative primary and secondary outcome measures, related-samples Wilcoxon signed-rank tests (baseline to post intervention) were conducted, and 95% confidence intervals were calculated by the Hodges–Lehman method.^41^ Analyses only included participants who completed assessments at both time points. Analyses were conducted in SPSS version 29 for macOS (IBM, Armonk, New York, USA; https://www.ibm.com/products/spss-statistic). CSQ-8 scores were analysed descriptively, and quantitative measures of feasibility were calculated as percentages. Effect sizes were calculated by transforming the *z*-statistic from Wilcoxon signed-rank tests into Cohen’s *r* (*r* = *z* 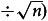 for interpretation of effect sizes, as recommended by Clark-Carter.^42^ Cohen’s^43^ criteria was used to interpret effect sizes, with Cohen’s *r* of 0.10 for a small effect, 0.30 for a medium effect and 0.50 for a large effect.

## Results

The participant flow is presented in Fig. 1. Parents registered between 7 April 2023 to 20 August 2023. Participants were followed up by the research team to complete post-intervention assessments between 16 August 2023 and 5 February 2024. Interviews were conducted between 31 August 2023 and 18 January 2024, and post-intervention assessments were collected between 16 August 2023 and 15 January 2024. The pilot trial concluded on 6 February 2024, when all participants completed or declined to complete their assessments or were lost to follow-up (i.e. no response to two attempts by the research team to follow up via SMS and email).

### Adverse events

Three serious adverse events occurred during the pilot trial, however, there were no deaths. Two adolescents of participating parents each had one suicide attempt, and another adolescent was admitted to an in-patient psychiatric facility following exacerbation of suicidal thoughts and self-injury. All three serious adverse events were deemed unrelated to the research as the adolescents’ suicidal thoughts and behaviour were a known risk before the trial, and all three adolescents had a history of suicide attempt before engagement in the trial. In all cases, parents continued to participate in the intervention.

### Programme acceptability, feasibility and validity

In terms of quantitative outcomes for programme acceptability, the mean score on the CSQ-8 was 30.77 (s.d. 1.74) out of a possible 32, indicating a high level of acceptability. Regarding programme feasibility, there were high programme completion rates. Of the selected modules parents intended to complete at the beginning of the programme, 96.20% were completed. Of the coaching sessions parents elected to attend, 93.98% were attended. For adherence, 93.33% of parents (*n* = 14) completed the 5 core modules, and the same 14 parents completed all 6 coaching sessions. Taken together, these results indicate that PiP-SP+ was feasible.

Further to quantitative outcomes, parents’ responses to the semi-structured, open-ended interview questions were coded by A.C. into the seven constructs of the TFA.^31^ Table 3 defines these seven constructs and presents parent participants’ responses, illustrated by indicative verbatim quotes. Subthemes were also identified inductively, including subthemes that did not fit within the TFA constructs but were still relevant to the general acceptability of PiP-SP+ (see Table 3 for parents’ quotes related to the programme’s acceptability, feasibility and validity).

**Table 3:** Parent reflections on the acceptability of PiP-SP+, including feasibility and validity

TFA construct: definition	Subtheme of TFA construct	Illustrative quote
Affective attitude (acceptability): ‘How an individual feels about the intervention’	Positive feedback	‘I was super impressed with it – like it’s awesome… I’m just so grateful that we had this opportunity to be a part of it.’ P5
	Challenging to provide feedback for improvement	‘I’m just trying to think if there was anything negative… oh, I wish I could give you something, but I can’t.’ P7
	Programme structure	‘I really like the structure of the programme. I really enjoyed the fact that there was a tailoring aspect to it and [coach] … it felt quite very cohesive.’ P10
	Confronting and emotionally challenging topic	‘Suicide and attempts at suicide, and your kid, your son, whoever your child is, saying that they don’t want to be here… It [PiP-SP+] was good. It was beneficial. But yeah, still, still tough.’ P6
Perceived effectiveness (validity): ‘The extent to which the intervention is perceived as likely to achieve its purpose’	Increased parental self-efficacy in responding to adolescent’s suicidal thoughts/behaviour	‘I can see things escalating and step in before it gets to that really suicidal stage if that makes sense. And then yeah, if she gets, if it does escalate to that point where I feel that she might be thinking along those lines, then I can step in, and I’m confident that I can say the right things and discuss it with her. Whereas before I, you know, was never sure.’ P4
	Increased parental self-efficacy in responding to adolescent’s non-suicidal self-injury	‘I was completely oblivious to self-harm and how it relates and so it [PiP-SP+] gave me a bit more confidence in terms of where is she at with it.’ P5
	Noticeable changes in parenting	‘The conversation that I then had with [adolescent], I run it so differently now… It was chalk and cheese to what would normally happen… it was amazing. At the end of the conversation [adolescent] was just like, ‘Oh, thanks Mum. That was really helpful”.’ P10
	Noticeable changes in adolescent	‘Self-harm has definitely been reduced a lot. … I find she’s more resilient at the moment, like just little things she’s done. I’m like, “okay, normally, she would have kind of fallen down in a situation like that“.’ P3
	Increased parent’s hope for their adolescent’s recovery	‘I felt like before I was always waiting for something bad to happen. And always being quite depressed about that… I’ve seen, you know, how I respond to things helps [adolescent] get over it and get, get over those crisis points, I do have a lot more hope now.’ P7
	Helped parent implementation of knowledge	‘I’ve heard a lot of stuff before. But I just wasn’t good at applying it at all, like, so I found it [the programme] was really good with my skill building.’ P8
	’Adolescent responses were unexpected and beyond parent’s control	‘I do think I know what to do, but then it’s the response from [adolescent] that is what throws things off… I could do that [PiP-SP+ strategy], but my child would not answer me back that way. You know, like no matter what would be said.’ P11
	Unable to attribute individual effects to PiP-SP	‘I don’t know if it’s the programme, or just how things have eventuated - that things have gotten better while we’re doing the programme. Look, it’s probably a combination of both.’ P3
Intervention coherence (validity): ‘Extent to which the participant understands the intervention and how it works’	Clear understanding of the intended effects of PiP-SP+	‘Everybody was always quite kind of clear on you know what it, what it was for and what it was going to be, and stuff like that.’ P2
	Parents were initially unsure of what to expect of programme	‘I don’t know if I had any expectations, to be honest, I didn’t know what to expect.’ P3
Ethicality: ‘Extent to which the intervention has a good fit with an individual’s value system’	Appropriate to parents	‘Absolutely appropriate with regards to all of those things… my whole yeah attitude towards parenting it definitely did fit.’ P4
	Relatable to parents	‘The content itself like there were a couple of times where it brought me to tears. I could really relate.’ P1
	Tailored to parents’ individuality	‘Sometimes I needed more time to stay with one area. But that was kind of something that we worked out between [coach] and I.’ P9
	Consider adaptation to underserved parents	‘Somebody who is vision impaired how would they engage? Someone who is hearing impaired, how would they engage? … I can imagine that parents with a disability just have, you know, one more layer of complexity in their life.’ P1
Burden (feasibility): ‘Perceived amount of effort that is required to participate in the intervention’	Effort was reasonable and worthwhile	‘It’s like anything that’s worth it. It takes an effort, but it’s worth it… It’s like a drop in the ocean compared to what benefits it can do - save your kid’s life. So, the effort, it is fine.’ P8
	Programme was flexible to parents’ needs	‘I thought the programme was very flexible. I didn’t sort of spend my time doing things that weren’t really relevant to me.’ P1
	At times challenging to complete with competing life demands	‘It wasn’t something that I wasn’t looking forward to and didn’t want to do or that it was arduous for me to do. It was just I just hadn’t found time in my week to do it.’ P5
Opportunity costs (feasibility): ‘Extent to which benefits, profits or values must be given up to engage in the intervention’	No opportunity costs, only benefits	‘It was opposite. You know I got from it rather than it took.’ P7
	Interfered with parents’ work commitments	‘What was very difficult for me was not being able to engage with the programme outside of work hours. I don’t know how a kind of person who works at the intensity that I work… to take time away from work on top of everything else.’ P1
	If a financial cost was associated, this would be challenging	‘If, you know, somebody had to pay to do this programme… Counselling, you know, privately is expensive enough. But if you want to try and help your family as well… I think if there was a cost involved, it probably might be difficult.’ P6
Confidence in completing programme^a^ (feasibility): ‘Participant’s confidence that they can perform the behaviour required to participate in the intervention’	Confidence in completing programme requirements	‘I was confident. I thought it would be fine. And I did complete it [PiP-SP+].’ P11
	May benefit from further opportunities for behaviour implementation	‘What came out was sometimes I needed more time to stay with one area… when you look at it for the time that you had to learn… have a coaching session about it, making goals, and then having the week to see how you can implement it… a week can go really fast. And you think “you know what? I haven’t really even tried to do that” or “I didn’t take any opportunities to do that”.’ P9
Additional acceptability subthemes	Parent’s hope for wider implementation of PiP-SP+	‘I would love to see this rolled out everywhere, to anyone who shows up in an emergency department with a teenager who is going through this crisis… Because, as I said, we got nothing when we went to the emergency department… It was really hard, and we were just floundering and not knowing what to do and where to go… I just wish that everyone who was going through the horrible time that we went through could have the same result.’ P5
	Parents could benefit from a follow-up booster session	‘A check-in would be something that would be really helpful… just tap in to go, “how are you going? Have you, have you been giving any new ones a go, or is there any questions you want to ask?” That might kind of just remind you to keep on track.’ P9
	Consider the inclusion of a secondary caregiver	‘There’s two parents, and it’s just one parent going to the programme. Getting the buy in from the other parent to go along with that goal sometimes can be difficult.’ P7
	Parents’ increased sense of advocacy and peer support	‘Doing that [the programme] also raises the awareness amongst the people I talk to as… It’s taboo when you start talking to people about it. But having done this programme, it enabled me to be up to talk about it with them.’ P7

TFA, Theoretical Framework of Acceptability; PiP-SP+, Partners in Parenting Plus – Suicide Prevention.

a. The construct ‘confidence in completing the programme’ was originally called ‘self-efficacy’ in the TFA.^31^ It was renamed here to clearly distinguish between parental self-efficacy in responding to their adolescent’s suicidal thoughts and behaviours and their self-efficacy in completing programme requirements.

#### Affective attitude

All parents expressed overwhelmingly positive attitudes towards PiP-SP+. They highlighted its usefulness in supporting them to navigate the challenges of parenting a suicidal adolescent. Parents appreciated the holistic approach of module content supplemented with coaching and a tailored Action Plan. Given the positive attitudes towards PiP-SP+, a number of parents described that it was difficult to provide feedback for improvement. A subset of parents noted that although they found the programme helpful, the topic of adolescent suicidality was emotionally taxing and confronting. However, this feedback reflected the challenging nature of the subject, rather than a critique of the programme itself.

#### Perceived effectiveness (validity)

Parents reported that the PiP-SP+ programme improved their self-efficacy in managing their adolescent’s suicidal thoughts, behaviours and non-suicidal self-injury. They noticed a positive shift in their parenting approach and their relationship with their adolescent. Additionally, parents reported observing a reduction in their adolescent’s suicidality and self-injury, which gave them hope for their adolescent’s recovery. Although parents found the programme helpful, some mentioned that all positive changes were not solely because of their participation in the PiP-SP+ programme, as other factors also contributed to their adolescent’s improvement (e.g. the adolescent’s own treatment, improvements in the school environment). Further, another subset of parents felt more confident in their actions, but struggled with not receiving the expected responses from their adolescents, and that their adolescents’ responses to the strategies were beyond their control.

#### Intervention coherence (validity)

Participants had a strong understanding of the PiP-SP+ programme and its benefits. However, despite being informed about the programme, parents initially felt uncertain about what they could expect from the programme.

#### Ethicality

Parents overwhelmingly found the programme appropriate and relatable to their individual circumstances. Many attributed this to the programme’s flexibility, which allowed coaches to tailor content to fit their unique needs. However, some parents noted that the programme might need adaptations for underserved groups, particularly those with disabilities or from culturally and linguistically diverse backgrounds.

#### Burden (feasibility)

Parents found the effort required to participate in the programme manageable and worthwhile, given the benefits they perceived. They appreciated the flexibility of completing module content at their convenience and negotiating coaching session times with their coach, which reduced the programme’s burden. However, some parents struggled to complete components of the programme because of competing life demands, particularly with work commitments and other unexpected life stressors.

#### Opportunity costs (feasibility)

Parents generally found participating in the programme worthwhile and did not perceive the need to make any sacrifices to participate. However, some noted that attending coaching sessions during business hours affected their work commitments. Additionally, although the pilot trial was cost-free, some parents described that it may be challenging to participate if fees were charged.

#### Confidence in completing the programme (feasibility)

Parents largely felt confident in completing all components of PiP-SP+, including the modules, coaching sessions and PiP-SP+ Action Plan. One area parents noted greater difficulty completing was the implementation of goals and associated parenting behaviour change. Parents reflected that they would have benefited from either increased opportunities to implement their behaviour change with the support of their coach and/or would benefit from having more time between sessions to complete their goals.

#### Additional acceptability subthemes

Parents expressed a strong desire for the PiP-SP+ programme to be more widely available, especially advertised via emergency departments, which they perceived would support other parents facing similar crises and could provide the guidance they felt was missing, based on their own experiences. Parents suggested adding booster sessions to help maintain their progress and allow continued opportunities to reflect on their parenting. Some parents also recommended adapting the programme to involve a second caregiver to align both parents with the programme’s goals. Finally, parents described that in following the programme, they perceived increases in their ability to advocate for and discuss adolescent suicide prevention, which in turn reduced stigma in the community and provided opportunities to support their parent peers.

### Baseline-to-post-intervention outcomes

Baseline-to-post intervention outcomes are presented in Tables 4 and 5 for parent- and adolescent-reported measures, respectively.

**Table 4:** Parent-reported quantitative outcomes: baseline-to-post-intervention outcomes (*n* = 13)

Outcome	Baseline mean (s.d.)	Baseline median	Post-intervention mean (s.d.)	Post-intervention median	95% CI of median difference^a^	*z*-value^b^	*p*-value	Cohen’s *r*
Parental self-efficacy to respond to adolescent suicidality	58.07 (17.14)	63.00	85.77 (8.88)	86.00	[21.50−37.50]	−3.18^c^	0.001	0.88
Parental self-efficacy to respond to adolescent non-suicidal self-injury	26.67 (9.06)	27.00	42.07 (5.27)	42.00	[11.50−21.50]	−3.18^c^	0.001	0.88
Parenting behaviours protective against adolescent anxiety and depression (PRADAS)	46.87 (3.40)	48.00	54.31 (5.92)	55.00	[4.50−11.00]	−2.98^c^	0.003	0.83
Parental self-efficacy to engage in protective parenting behaviours (PSES)	21.47 (4.47)	20.00	27.38 (3.55)	29.00	[3.50−9.50]	−3.06^c^	0.003	0.85
Quality of mental health support intended to be provided (MHSS-I)	63.27 (5.73)	63.00	76.23 (6.02)	75.00	[11.00−18.00]	−3.19^c^	0.001	0.89
Carer burden (BAS)	59.33 (7.51)	60.00	51.46 (13.39)	53.00	[−17.00 to 0.00]	−2.09^c^	0.036	0.58
Family functioning (FAD-GF)	25.33 (3.18)	25.00	23.46 (4.68)	24.00	[−4.00 to 0.50]	−1.61^c^	0.106	0.45
Parent psychological distress (K6)	15.07 (4.53)	16.00	12.31 (5.12)	13.00	[−6.00 to 0.00]	−1.97^d^	0.049	0.54
Parent-report of their adolescent’s anxiety symptoms(RCADS-25-P anxiety subscale)	15.47 (5.76)	15.00	10.69 (5.20)	10.00	[−7.00 to −1.50]	−2.59^d^	0.010	0.72
Parent-report of their adolescent’s depressive symptoms (RCADS-25-P depressive subscale)	17.60 (3.74)	17.00	13.54 (4.35)	14.00	[−7.00 to −1.00]	−2.51^d^	0.012	0.69

PRADAS, Parenting to Reduce Adolescent Depression and Anxiety Scale; PSES, Parental Self-Efficacy Scale; MHSS-I, Mental Health Support Scale – Intended; BAS, Burden Assessment Scale; FAD-GF, McMaster Family Assessment Device, General Functioning subscale; K6, Kessler-6 scale; RCADS-25-P, Revised Child Anxiety and Depression Scale 25-item – Parent Version.

a. The 95% CI represents the upper and lower bound of the median difference, as calculated by the related-samples Hodges−Lehman method.^41^.

b. Related-samples Wilcoxon signed-rank test, standardised test statistic.

c. Based on negative ranks.

d. Based on positive ranks.

**Table 5:** Adolescent-reported quantitative outcomes: pre–post intervention outcomes (*n* = 9)

Outcome	Baseline mean (s.d.)	Baseline median	Post-intervention mean (s.d.)	Post-intervention median	95% CI of median difference^a^	*z*-value^b^	*p*-value	*r*
Perceived parental emotional support if the adolescent was experiencing suicidal ideation or self-injuring	9.67 (3.57)	10.00	11.78 (2.77)	12.00	[0.00−4.00]	−2.03^c^	0.042	0.68
Adolescent self-reported anxiety symptoms (RCADS-25 anxiety subscale)	21.33 (5.70)	21.00	14.67 (6.28)	14.00	[−11.00 to −3.50]	−2.67^d^	0.008	0.89
Adolescent self-reported depressive symptoms (RCADS-25 depressive subscale)	17.67 (4.74)	18.00	16.22 (3.87)	16,00	[−6.50 to 1.00]	−1.19^d^	0.235	0.40

RCADS-25, Revised Child Anxiety and Depression Scale 25-item version.

a. The 95% CI represents the upper and lower bound of the median difference, as calculated by the related-samples Hodges−Lehman method.^41^.

b. Related-samples Wilcoxon signed ranks test, standardised test statistic.

c. Based on negative ranks.

d. Based on positive ranks.

As shown in Table 4, there were statistically significant improvements from pre- to post-intervention, with large effect sizes in parental self-efficacy to respond to adolescent suicidality, parental self-efficacy to respond to adolescent non-suicidal self-injury, parenting behaviours protective against adolescent anxiety and depression, parental self-efficacy to engage in protective parenting behaviours, quality of mental health support intended to be provided, carer burden, psychological distress, parent-report of adolescent anxiety symptoms and parent-report of adolescent depressive symptoms. A statistically significant change in family functioning was not detected. Similarly, as shown in Table 5, adolescent-reported data showed statistically significant improvements, with large effect sizes for perceived parental emotional support and adolescent self-reported anxiety symptoms. No significant change was detected in adolescent self-reported depressive symptoms.

## Discussion

To our knowledge, PiP-SP+ is the first individually tailored, coach-supported online parenting programme for parents of adolescents experiencing suicidal thoughts or behaviours. This study demonstrated the acceptability, feasibility and validity of the PiP-SP+ intervention. Although promising patterns of change were observed across several outcomes, these baseline-to-post-intervention findings should be interpreted with caution because of the absence of a control group and small sample size. Parents reported significant increases in their self-efficacy in responding to adolescent suicidality and non-suicidal self-injury, and provided corroborating qualitative accounts. Additionally, significant improvements were observed in parent-reported parenting behaviours protective against adolescent anxiety and depression, quality of mental health support given by parents to their adolescents and carer burden. Adolescents reported perceiving increased parental emotional support if they were suicidal or self-injuring, and both parents and adolescents reported reductions in adolescent anxiety symptoms. Although parents reported significant reductions in adolescent depressive symptoms, adolescents did not report this improvement themselves. Further, no significant change was detected in parents’ reports of family functioning.

### Acceptability

Overall, parents appeared to find the programme highly acceptable, providing overwhelmingly positive feedback. The programme’s strong alignment with the needs of the parent population may be attributed to the co-design process, where the end-users’ needs were considered throughout the intervention development process.^27^ Although this study did not assess adolescent suicide-related outcomes, the observed increase in parents’ self-efficacy is noteworthy, as it has been associated with lower future adolescent suicidality.^8^ Additionally, parents emphasised the value of the programme’s tailored structure. These findings echo the body of research underscoring the necessity of evidence-based, individually tailored parenting interventions for adolescent suicide prevention interventions.^44^ Despite finding the content emotionally taxing, parents who completed the PiP-SP+ reported a significant increase in self-efficacy in responding to suicidality and a notable decrease in carer burden. This contrasts with previous literature that highlights parents’ persistent distress owing to inadequate support.^45,46^ Although participants did experience some distress, they also emphasised that the benefits of the programme far outweighed these effects.

### Feasibility

The high adherence and programme completion rates, alongside the qualitative data, endorse the feasibility of completing the programme. Parents’ accounts suggest that such feasibility rates could be attributed to the flexible nature of the PiP-SP+, particularly the completion of modules at a time of their convenience. Study findings are consistent with prior research, which demonstrates that parents prefer engaging with online parenting programmes because of their flexible nature and reduced logistical barriers typically encountered with face-to-face interventions.^10^ Notably, 93.33% of parents completed the five core modules, and the same 14 parents completed all six coaching sessions. This level of adherence exceeds the average retention rate of 86.5% reported in a systematic review of technology-assisted parenting programmes.^47^ Although feasibility was high, as evidenced by completion and adherence rates, findings suggested the programme could be improved by providing more opportunities for parents to implement and reflect on their behaviour changes. A subset of participants noted that a week may be insufficient time for parents to learn and implement new parenting strategies. The feasibility of the PiP-SP+ could be further improved by accommodating coaching sessions outside of business hours, to minimise work disruptions for employed parents. Although the feasibility of the PiP-SP+ could potentially be enhanced by offering coaching sessions outside of business hours to minimise work disruptions, this approach raises feasibility and affordability challenges for service providers, which would need to be considered in future. Additionally, some parents requested booster sessions to maintain adaptive parenting strategies developed during the programme. Existing research provides little support for the efficacy of booster sessions in parenting interventions,^48^ but booster sessions for parents of suicidal adolescents may be critical and salient for continuity of care, particularly as these parents often report feeling isolated and lack sufficient professional support during their adolescent’s crises.^46^

### Validity

The PiP-SP+ programme demonstrated design validity as parents reported having a clear understanding of the intended effects of the PiP-SP+. However, parents expressed initial uncertainty regarding what they could expect from the programme. This finding is unsurprising, given that there are no other resources that offer parents intensive coaching support for adolescent suicide prevention,^49^ and there are generally limited supports available for parents following an adolescent suicidal crisis.^49^ The limited support described by parents before engaging in the PiP-SP+ programme underscores their hopes for the programme’s broader implementation. Further, parents revealed that they were able to discuss their experiences of parenting an adolescent experiencing suicidality more openly with other parents. They recounted this was an unintended but rewarding outcome of the PiP-SP+ intervention. As such, this finding aligns with the principles of peer-led parenting interventions, highlighting the value of mutual understanding and empathy among parents facing similar challenges.^50,51^

### Safety of the trial

Serious adverse events that occurred during the trial were deemed to be unrelated to the study protocol; hence, the lack of trial-related adverse events suggests that the programme did not do any harm. In addition to formally reported adverse events, adolescent symptoms of anxiety and depression and parent psychological distress were monitored from baseline to post-intervention, because, as well as being outcome measures, they may also offer some indication of whether the programme was associated with any unintended negative psychological effects. Overall, following the trial, there was a reduction in adolescent-reported anxiety symptoms and no changes were observed in adolescent-reported of depressive symptoms. Additionally, there was no significant increase in parental psychological distress observed following the intervention. Together, such findings support the psychological safety of the programme for both parents and their adolescents.

### Parent-reported baseline-to-post-intervention outcomes

Study findings largely mirror the short-term effects found in the original PiP+ programme.^15^ Both interventions improved protective parenting behaviours against anxiety and depression and enhanced parental self-efficacy in these areas. Notably, the current study also found self-reported improvements in parental self-efficacy to respond to adolescent suicidality and non-suicidal self-injury following the PiP-SP+ intervention. Given the improvements in protective parenting behaviours, it is unsurprising that the quality of mental health support parents provided to their adolescents (i.e. general knowledge and skills needed to recognise and respond to mental health problems) also improved following PiP-SP+. Although no significant reductions in carer burden were found in the original PiP+ trial,^15^ our study found significant improvements in carer burden and parental distress after completing PiP-SP+. This finding may be attributed to the greater emphasis on parental self-care in the PiP-SP+ programme (compared with the original PiP+ programme), because self-care content was added based on findings during the PiP-SP+ co-design process.^29^ Unlike PiP+, where no significant changes were reported in parent or adolescent-report of anxiety and depressive symptoms,^15^ PiP-SP+ showed concordant reports of improvements in both parent- and adolescent-reports of anxiety. However, although parents reported reduced depressive symptoms in their adolescents following the PiP-SP+ intervention, adolescents did not.

Notably, these results, and particularly the observed effect sizes, should be interpreted with caution given the study’s limited statistical power and the absence of a control group, which preclude definitive conclusions about efficacy. Although PiP+ was evaluated with some control for the natural course of decrease in symptoms through employing a double baseline,^20^ PiP-SP+ was not. Additionally, as adolescents were also engaged in professional mental healthcare, the effects of PiP-SP+ on their symptoms cannot be isolated from any treatment received.

There was no evidence of significant change in family functioning following the PiP-SP+ programme. Although there were improvements in parental factors, such as parental self-efficacy in managing adolescent suicidality, these effects were not observed in family functioning. This may be because family functioning is a more distally related outcome compared to the more immediate parental factors, which PiP-SP+ targets. Alternatively, this finding may highlight a potential limitation of the PiP-SP+ programme in that it is primarily designed for one parent. As suggested by some parents, expanding the programme to involve both parents could potentially enhance its effects on family functioning.

### Adolescent-reported outcomes

In addition to short-term effects reported by parents, adolescents perceived an increase in emotional support from parents if they were experiencing suicidal ideation or self-injuring. However, it is important to note that some qualitative feedback from parents indicates that not all adolescents responded to the changes in parenting behaviours as parents anticipated. Since the assessment did not include all adolescents from participating families, the findings may not fully represent the varied responses of adolescents to the PiP-SP+ programme.

### Future directions

Findings from the current trial highlight various future directions for the PiP-SP+. First, despite existing research providing little support for the efficacy of booster sessions, in parenting interventions,^48^ a booster session a few weeks post-intervention may critical and salient for continuity of care; particularly as these parents often report feeling isolated and lack sufficient professional support during their adolescent’s crises.^52^ A booster session with coaches could support parents to reinforce parenting strategies and provide parents with emotional support and validation from a coach with whom they have already built therapeutic rapport. Second, as suggested by some parents, the programme could be enhanced by involving both parents, thus future iterations of PiP-SP+ could explore ways to support the coach to deliver the programme to multiple caregivers. Third, although PiP-SP+ was perceived as acceptable, feasible and valid for parents, future adaptations are needed for underserved and underrepresented groups, such as parents with disabilities (e.g. providing a customisable technology interface to change font size or contrast). Additionally, PiP-SP+ could benefit from cultural adaptations to enhance its relevance for culturally and linguistically diverse communities. Finally, parents expressed a strong desire for the PiP-SP+ programme to be more widely available, especially via emergency departments. Thus, should PiP-SP+ demonstrate efficacy in an appropriately powered, randomised clinical trial, parents could be referred to PiP-SP+ from accident and emergency departments, which is where these families in crisis may present.

### Limitations

When interpreting the findings of this study, its limitations should be considered. First, because of the study design, we cannot distinguish between effects attributable to PiP-SP+ and those influenced by other confounding factors (e.g. adolescent mental health treatment, spontaneous recovery). Second, adolescent suicidality was not assessed because of feasibility constraints of the research team and to reduce participant burden; however, this limits the understanding of the effects of PiP-SP+ on this outcome. Third, the parent sample was predominantly Australian/New Zealand mothers, female, highly educated and engaged in the intervention. Although the limited uptake of PiP-SP+ among fathers is consistent with low rates of father participation reported in the broader parenting intervention literature,^53,54^ the recruitment material across the study was generally targeted toward ‘parents’. Recruitment material that separately calls for fathers may have potentially increased fathers’ engagement.^55^ Additionally, some parents declined to participate in the post-intervention assessment, which may have potentially positively biased parent feedback. Consequently, the available qualitative data on acceptability, feasibility and validity may not provide a comprehensive representation of all participants’ experiences, which may limit transferability. Fourth, PiP-SP+ is intensive and time-consuming, often spanning over 14 weeks. Its personalised, intensive and comprehensive delivery may therefore exceed resources available in some health care services. Fifth, the internal consistency of some measures, specifically the adolescent-report outcome measures, is lower than the conventionally accepted threshold of Cronbach’s *α* = 0.70, specifically the adolescent-report outcome measures. Consequently, these results should be interpreted with caution. Yet, given the mixed-methodology in which qualitative feedback is consistent with quantitative findings, the overall trends observed in the study may still offer valuable insights into the effects of the PiP-SP+ programme.

In conclusion, the mixed-methods findings of the PiP-SP+ pilot study suggest that the intervention is acceptable, feasible and valid. Such findings support the value of undertaking an appropriately powered, randomised controlled trial to evaluate intervention efficacy. The PiP-SP+ programme fills a critical research and resource gap by supporting parents of adolescents facing suicidality, and ultimately, it is hoped that such an intervention can prevent adolescent suicide by empowering parents with the skills and confidence needed to intervene.

## Supporting information

10.1192/bjo.2026.11046.sm001 Cao et al. supplementary material Cao et al. supplementary material

## Data Availability

A summary of the data that support the findings of this study are available on request from the corresponding author, M.B.H.Y. The data are not publicly available owing to their containing information that could compromise the privacy of research participants.

## Supplementary material

The supplementary material is available online at https://doi.org/10.1192/bjo.2026.11046

## References

[1] Kessler RC , Angermeyer M , Anthony JC , Graaf RD , Gasquet I , Girolamo GD , et al. Lifetime prevalence and age-of-onset distributions of mental disorders in the World Health Organization’s World Mental Health Survey Initiative. World Psychiatry 2007; 6: 168–76. 18188442 PMC2174588

[2] Gili M , Castellví P , Vives M , de la Torre-Luque A , Almenara J , Blasco MJ , et al. Mental disorders as risk factors for suicidal behavior in young people: a meta-analysis and systematic review of longitudinal studies. J Affect Disord 2019; 245: 152–62. 30390504 10.1016/j.jad.2018.10.115

[3] Michelmore L , Hindley P. Help-seeking for suicidal thoughts and self-harm in young people: a systematic review. Suicide Life Threat Behav 2012; 42: 507–24. 22889130 10.1111/j.1943-278X.2012.00108.x

[4] Brent DA , McMakin DL , Kennard BD , Goldstein TR , Mayes TL , Douaihy AB. Protecting adolescents from self-harm: a critical review of intervention studies. J Am Acad Child Adolesc Psychiatry 2013; 52: 1260–71. 24290459 10.1016/j.jaac.2013.09.009 PMC3873716

[5] Glenn CR , Franklin JC , Nock MK. Evidence-based psychosocial treatments for self-injurious thoughts and behaviors in youth. J Clin Child Adolesc Psychol 2015; 44: 1–29. 25256034 10.1080/15374416.2014.945211 PMC4557625

[6] King CA , Hill RM , Wynne HA , Cunningham RM. Adolescent suicide risk screening: the effect of communication about type of follow-up on adolescents’ screening responses. J Clin Child Adolesc Psychol 2012; 41: 508–15. 22540534 10.1080/15374416.2012.680188 PMC3790145

[7] Yap MBH , Reavley N , Jorm AF. Where would young people seek help for mental disorders and what stops them? Findings from an Australian national survey. J Affect Disord 2013; 147: 255–61. 23228570 10.1016/j.jad.2012.11.014

[8] Czyz EK , Horwitz AG , Yeguez CE , Ewell Foster CJ , King CA. Parental self-efficacy to support teens during a suicidal crisis and future adolescent emergency department visits and suicide attempts. J Clin Child Adolesc Psychol 2018; 47: S384–96. 28715239 10.1080/15374416.2017.1342546

[9] Hooven C. Parents-CARE: a suicide prevention program for parents of at-risk youth. J Child Adolesc Psychiatr Nurs 2013; 26: 85–95. 23351111 10.1111/jcap.12025

[10] Baker S , Sanders MR , Morawska A. Who uses online parenting support? A cross-sectional survey exploring Australian parents’ internet use for parenting. J Child Fam Stud 2017; 26: 916–27.

[11] Flujas-Contreras JM , García-Palacios A , Gómez I. Technology-based parenting interventions for children’s physical and psychological health: a systematic review and meta-analysis. Psychol Med 2019; 49: 1787–98. 30977462 10.1017/S0033291719000692

[12] Cao A , Melvin GA , Wu L , Cardamone-Breen MC , Salvaris CA , Olivier P , et al. Understanding the lived experience and support needs of parents of suicidal adolescents to inform an online parenting programme: qualitative study. BJPsych Open 2025; 11: e61. 40103508 10.1192/bjo.2024.855 PMC12001958

[13] Fulgoni CMF , Melvin GA , Jorm AF , Lawrence KA , Yap MBH. The Therapist-assisted Online Parenting Strategies (TOPS) program for parents of adolescents with clinical anxiety or depression: development and feasibility pilot. Internet Interv 2019; 18: 100285. 31890632 10.1016/j.invent.2019.100285 PMC6926173

[14] Yap MBH , Lawrence KA , Rapee RM , Cardamone-Breen MC , Green J , Jorm AF. Partners in parenting: a multi-level web-based approach to support parents in prevention and early intervention for adolescent depression and anxiety. JMIR Ment Health 2017; 4: e59. 29258974 10.2196/mental.8492 PMC5750418

[15] Khor SPH , Fulgoni CM , Lewis D , Melvin GA , Jorm AF , Lawrence K , et al. Short-term outcomes of the Therapist-assisted Online Parenting Strategies intervention for parents of adolescents treated for anxiety and/or depression: a single-arm double-baseline trial. Aust N Z J Psychiatry 2022; 56: 695–708. 34231423 10.1177/00048674211025695

[16] Campbell M , Fitzpatrick R , Haines A , Kinmonth AL , Sandercock P , Spiegelhalter D , et al. Framework for design and evaluation of complex interventions to improve health. BMJ 2000; 321: 694–6. 10987780 10.1136/bmj.321.7262.694 PMC1118564

[17] Craig P , Dieppe P , Macintyre S , Michie S , Nazareth I , Petticrew M. Developing and evaluating complex interventions: the new Medical Research Council guidance. BMJ 2008; 337: a1655. 18824488 10.1136/bmj.a1655 PMC2769032

[18] Creswell JW , Plano Clark VL. Designing and Conducting Mixed Methods Research. SAGE Publications, 2007.

[19] Eldridge SM , Chan CL , Campbell MJ , Bond CM , Hopewell S , Thabane L , et al. CONSORT 2010 statement: extension to randomised pilot and feasibility trials. BMJ 2016; 355: i5239. 27777223 10.1136/bmj.i5239 PMC5076380

[20] Birkett MA , Day SJ. Internal pilot studies for estimating sample size. Stat Med 1994; 13: 2455–63. 7701146 10.1002/sim.4780132309

[21] Chorpita BF , Ebesutani C , Spence SH . *Revised Child Anxiety and Depression Scale: User’s Guide*. University of California, Los Angeles, 2022 (https://rcads.ucla.edu/).

[22] Ebesutani C , Korathu-Larson P , Nakamura BJ , Higa-McMillan C , Chorpita B. The Revised Child Anxiety and Depression Scale 25-parent version: scale development and validation in a school-based and clinical sample. Assessment 2017; 24: 712–28. 26834091 10.1177/1073191115627012

[23] Ebesutani C , Reise SP , Chorpita BF , Ale C , Regan J , Young J , et al. The Revised Child Anxiety and Depression Scale-Short Version: scale reduction via exploratory bifactor modeling of the broad anxiety factor. Psychol Assess 2012; 24: 833–45. 22329531 10.1037/a0027283

[24] Harris PA , Taylor R , Thielke R , Payne J , Gonzalez N , Conde JG. Research electronic data capture (REDCap) – a metadata-driven methodology and workflow process for providing translational research informatics support. J Biomed Inform 2009; 42: 377–81. 18929686 10.1016/j.jbi.2008.08.010 PMC2700030

[25] Harris PA , Taylor R , Minor BL , Elliott V , Fernandez M , O’Neal L , et al. The REDCap consortium: building an international community of software platform partners. J Biomed Inform 2019; 95: 103208. 31078660 10.1016/j.jbi.2019.103208 PMC7254481

[26] Cardamone-Breen MC , Jorm AF , Lawrence KA , Mackinnon AJ , Yap MBH. The Parenting to Reduce Adolescent Depression and Anxiety Scale: assessing parental concordance with parenting guidelines for the prevention of adolescent depression and anxiety disorders. PeerJ 2017; 5: e3825. 28951815 10.7717/peerj.3825 PMC5609518

[27] Design Council. History of the Double Diamond . Design Council, 2024 (https://www.designcouncil.org.uk/our-resources/the-double-diamond/history-of-the-double-diamond/).

[28] Ross AM , Kelly CM , Jorm AF. Re-development of mental health first aid guidelines for suicidal ideation and behaviour: a Delphi study. BMC Psychiatry 2014; 14: 241. 25213799 10.1186/s12888-014-0241-8 PMC4199061

[29] Cao A, Wu L, Melvin G, Cardamone-Breen M, Broomfield G, Seguin J, et al. Empowering parents of adolescents at elevated risk of suicide: co-designing an adaptation to a coach-assisted, digital parenting intervention. *Eur J Investig Health Psychol Educ* 2025; 15: 199. 10.3390/ejihpe15100199 PMC12563294 41149150

[30] Larsen DL , Attkisson CC , Hargreaves WA , Nguyen TD. Assessment of client/patient satisfaction: development of a general scale. Eval Program Plann 1979; 2: 197–207. 10245370 10.1016/0149-7189(79)90094-6

[31] Sekhon M , Cartwright M , Francis JJ. Development of a theory-informed questionnaire to assess the acceptability of healthcare interventions. BMC Health Serv Res 2022; 22: 279. 35232455 10.1186/s12913-022-07577-3 PMC8887649

[32] Sekhon M , Cartwright M , Francis JJ. Acceptability of healthcare interventions: an overview of reviews and development of a theoretical framework. BMC Health Serv Res 2017; 17: 88. 28126032 10.1186/s12913-017-2031-8 PMC5267473

[33] Parenting Strategies Program. How to Prevent Depression and Clinical Anxiety in Your Teenager: Strategies for Parents. Beyondblue, 2013 (https://www.parentingstrategies.net/).

[34] Subkoviak MJ. A practitioner’s guide to computation and interpretation of reliability indices for mastery tests. J Educ Meas 1988; 25: 47–55.

[35] Nicolas CC , Jorm AF , Cardamone-Breen MC , Lawrence KA , Yap MBH. Parental self-efficacy for reducing the risk of adolescent depression and anxiety: scale development and validation. J Res Adolesc 2020; 30: 249–65. 31246378 10.1111/jora.12521

[36] Morgan AJ , Wright J , Mackinnon AJ , Reavley NJ , Rossetto A , Jorm AF. Development of the mental health support scale: a new measure of mental health first aid behaviors. Assessment 2023; 30: 1486–98. 35758161 10.1177/10731911221106767

[37] Reinhard SC , Gubman GD , Horwitz AV , Minsky S. Burden assessment scale for families of the seriously mentally ill. Eval Program Plann 1994; 17: 261–9.

[38] Epstein NB , Baldwin LM , Bishop DS. The McMaster family assessment device. J Marital Fam Ther 1983; 9: 171–80.

[39] Kessler RC , Barker PR , Colpe LJ , Epstein JF , Gfroerer JC , Hiripi E , et al. Screening for serious mental illness in the general population. Arch Gen Psychiatry 2003; 60: 184–9. 12578436 10.1001/archpsyc.60.2.184

[40] Braun V , Clarke V. Thematic Analysis: A Practical Guide. SAGE London, 2022.

[41] Hodges JLJ , Lehmann EL. Estimates of location based on rank tests. Ann Math Stat 1963; 34: 598–611.

[42] Clark-Carter D. Quantitative Psychological Research: The Complete Student’s Companion 3rd ed. Routledge, 2019.

[43] Cohen J. Statistical Power Analysis for the Behavioral Sciences 2nd ed. Routledge, 1988.

[44] Watling DP , Preece MHW , Hawgood J , Bloomfield S , Kõlves K. Developing a post-discharge suicide prevention intervention for children and young people: a qualitative study of integrating the lived-experience of young people, their carers, and mental health clinicians. Child Adolesc Psychiatry Ment Health 2022; 16: 24. 35346301 10.1186/s13034-022-00460-3 PMC8958759

[45] Lachal J , Orri M , Sibeoni J , Moro MR , Revah-Levy A. Metasynthesis of youth suicidal behaviours: perspectives of youth, parents, and health care professionals. PLOS One 2015; 10: e0127359. 26001066 10.1371/journal.pone.0127359 PMC4441448

[46] Weissinger GM , Evans L , Van Fossen C , Winston-Lindeboom P , Ruan-Iu L , Rivers RS. Parent experiences during and after adolescent suicide crisis: a qualitative study. Int J Mental Health Nurs 2023; 32: 917–28. 10.1111/inm.13137 36882964

[47] Hansen A , Broomfield G , Yap MBH. A systematic review of technology-assisted parenting programs for mental health problems in youth aged 0-18 years: applicability to underserved Australian communities. Austr J Psychol 2019; 71: 433–62.

[48] Eyberg S , Boggs S , Jaccard J. Does maintenance treatment matter? J Abnorm Child Psychol 2014; 42: 355–66. 24413969 10.1007/s10802-013-9842-9

[49] Torok M , Calear AL , Smart A , Nicolopoulos A , Wong Q. Preventing adolescent suicide: a systematic review of the effectiveness and change mechanisms of suicide prevention gatekeeping training programs for teachers and parents. J Adolesc 2019; 73: 100–12. 31054373 10.1016/j.adolescence.2019.04.005

[50] Day C , Michelson D , Thomson S , Penney C , Draper L. Innovations in practice: empowering parents, empowering communities: a pilot evaluation of a peer-led parenting programme. Child Adoles Ment Health 2012; 17: 52–7. 10.1111/j.1475-3588.2011.00619.x 32847315

[51] Wu L , Seguin JP , Chandrasekara D , Cardamone-Breen MC , Xie J , Mcnaney R , et al. Designing online peer support for parents of adolescents at risk of mental health challenges. 2024 CHI Conference on Human Factors in Computing Systems (Honolulu, USA, 11–16 May 2024). Association for Computing Machinery, 2024.

[52] Rheinberger D , Shand F , Mcgillivray L , Mccallum S , Boydell K. Parents of adolescents who experience suicidal phenomena – a scoping review of their experience. Int J Environ Res Public Health 2023; 20: 6227. 37444075 10.3390/ijerph20136227 PMC10340647

[53] Sicouri G , Tully L , Collins D , Burn M , Sargeant K , Frick P , et al. Toward father-friendly parenting interventions: a qualitative study. Austr N Z J Family Ther 2018; 39: 218–31. 10.1002/anzf.1307 PMC6033039 30008513

[54] Gonzalez JC , Klein CC , Barnett ML , Schatz NK , Garoosi T , Chacko A , et al. Intervention and implementation characteristics to enhance father engagement: a systematic review of parenting interventions. Clin Child Fam Psychol Rev 2023; 26: 445–58. 36947287 10.1007/s10567-023-00430-x PMC10031187

[55] Hansen A , Wade C , Yap MB. Fathers’ perspectives on engaging with web-based parenting programs for adolescent mental health: a qualitative study. Mental Health Prevent 2022; 26: 200232.

