# Supplementary material for: A coach-supported, digital parenting programme for parents of adolescents at risk of suicide: pilot trial of acceptability, feasibility, validity and short-term effects
Source: BJPsych Open. 2026 May 19;12(3):e138. doi: 10.1192/bjo.2026.11046 (PMC13184607; doi:10.1192/bjo.2026.11046)
Supplement: Cao et al. supplementary material [file S2056472426110461sup001.docx]

**Supplementary Material A: CONSORT checklist**

**CONSORT 2010 checklist of information to include when reporting a pilot or feasibility trial***
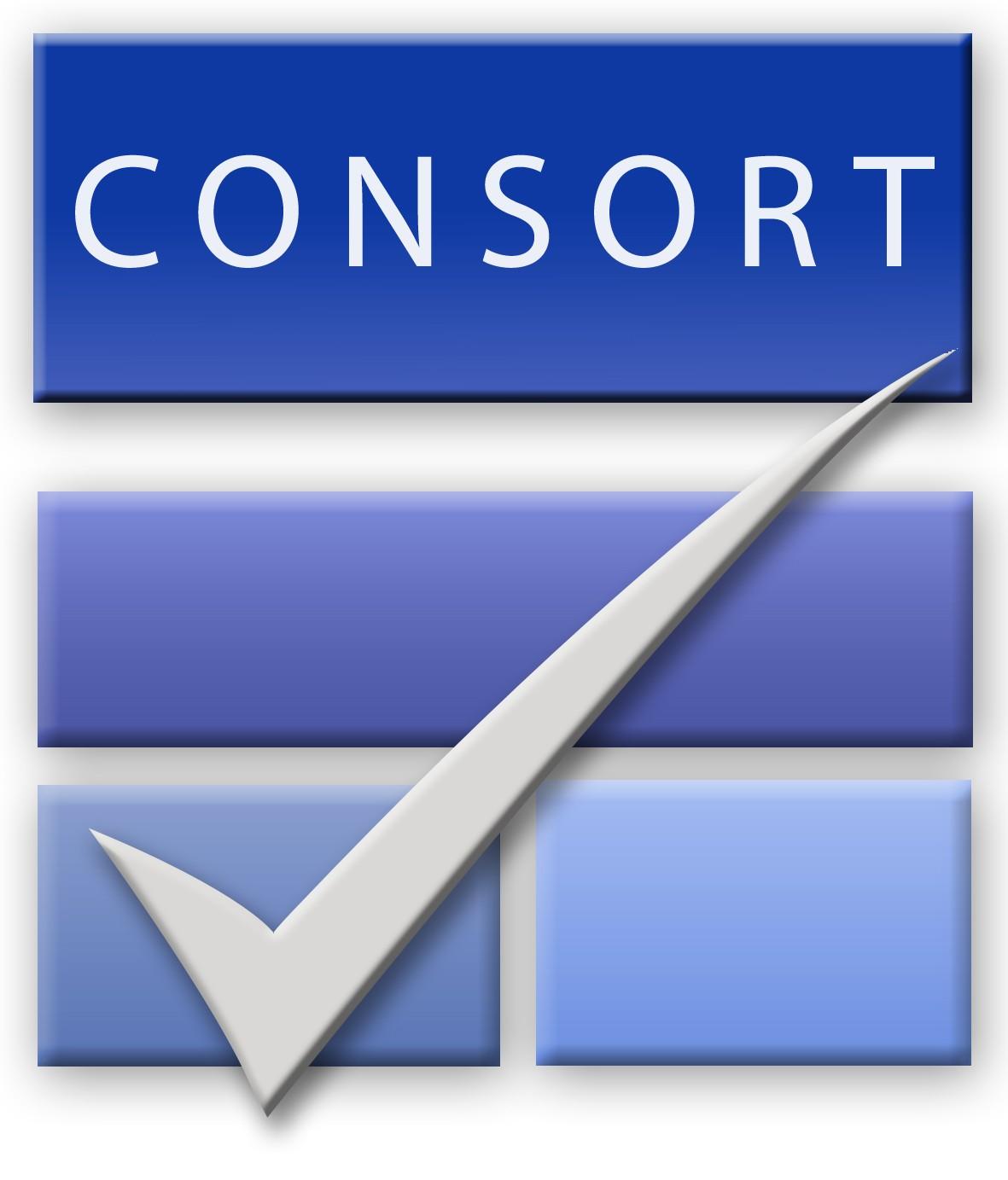


| **Section/Topic** | **Item No** | **Checklist item** | **Reported on page No** |
| --- | --- | --- | --- |
| **Title and abstract** | | | |
|  | 1a | Identification as a pilot or feasibility randomised trial in the title | 1 |
|  | 1b | Structured summary of pilot trial design, methods, results, and conclusions (for specific guidance see CONSORT abstract extension for pilot trials) | 2 |
| **Introduction** | | | |
| Background and objectives | 2a | Scientific background and explanation of rationale for future definitive trial, and reasons for randomised pilot trial | 4-6 |
|  | 2b | Specific objectives or research questions for pilot trial | 6 |
| **Methods** | | | |
| Trial design | 3a | Description of pilot trial design (such as parallel, factorial) including allocation ratio | 6,7 |
|  | 3b | Important changes to methods after pilot trial commencement (such as eligibility criteria), with reasons | 7 |
| Participants | 4a | Eligibility criteria for participants | 7 |
|  | 4b | Settings and locations where the data were collected | 11,12 |
|  | 4c | How participants were identified and consented | 7, 11, 12 |
| Interventions | 5 | The interventions for each group with sufficient details to allow replication, including how and when they were actually administered | 12-14 |
| Outcomes | 6a | Completely defined prespecified assessments or measurements to address each pilot trial objective specified in 2b, including how and when they were assessed | 14-17 |
|  | 6b | Any changes to pilot trial assessments or measurements after the pilot trial commenced, with reasons | 7 |
|  | 6c | If applicable, prespecified criteria used to judge whether, or how, to proceed with future definitive trial | 5,6 |
| Sample size | 7a | Rationale for numbers in the pilot trial | 7 |
|  | 7b | When applicable, explanation of any interim analyses and stopping guidelines | Not applicable |
| Randomisation: |  |  |  |
| Sequence  generation | 8a | Method used to generate the random allocation sequence | Not applicable |
|  | 8b | Type of randomisation(s); details of any restriction (such as blocking and block size) | Not applicable |
| Allocation  concealment  mechanism | 9 | Mechanism used to implement the random allocation sequence (such as sequentially numbered containers), describing any steps taken to conceal the sequence until interventions were assigned | Not applicable |
| Implementation | 10 | Who generated the random allocation sequence, who enrolled participants, and who assigned participants to interventions | Not applicable |
| Blinding | 11a | If done, who was blinded after assignment to interventions (for example, participants, care providers, those assessing outcomes) and how | Not applicable |
|  | 11b | If relevant, description of the similarity of interventions | Not applicable |
| Statistical methods | 12 | Methods used to address each pilot trial objective whether qualitative or quantitative | 18 |
| **Results** | | | |
| Participant flow (a diagram is strongly recommended) | 13a | For each group, the numbers of participants who were approached and/or assessed for eligibility, randomly assigned, received intended treatment, and were assessed for each objective | 10 |
|  | 13b | For each group, losses and exclusions after randomisation, together with reasons | 10 |
| Recruitment | 14a | Dates defining the periods of recruitment and follow-up | 19 |
|  | 14b | Why the pilot trial ended or was stopped | 19 |
| Baseline data | 15 | A table showing baseline demographic and clinical characteristics for each group | 7 - 10 |
| Numbers analysed | 16 | For each objective, number of participants (denominator) included in each analysis. If relevant, these numbers should be by randomised group | 10 |
| Outcomes and estimation | 17 | For each objective, results including expressions of uncertainty (such as 95% confidence interval) for any estimates. If relevant, these results should be by randomised group | 27 - 29 |
| Ancillary analyses | 18 | Results of any other analyses performed that could be used to inform the future definitive trial | 23 - 25 Qualitative results |
| Harms | 19 | All important harms or unintended effects in each group (for specific guidance see CONSORT for harms) | 33 |
|  | 19a | If relevant, other important unintended consequences | Not applicable |
| **Discussion** | | | |
| Limitations | 20 | Pilot trial limitations, addressing sources of potential bias and remaining uncertainty about feasibility | 36-37 |
| Generalisability | 21 | Generalisability (applicability) of pilot trial methods and findings to future definitive trial and other studies | 36 |
| Interpretation | 22 | Interpretation consistent with pilot trial objectives and findings, balancing potential benefits and harms, and considering other relevant evidence | 33 |
|  | 22a | Implications for progression from pilot to future definitive trial, including any proposed amendments | 35-36 |
| **Other information** | | |  |
| Registration | 23 | Registration number for pilot trial and name of trial registry | 6 |
| Protocol | 24 | Where the pilot trial protocol can be accessed, if available | 6 |
| Funding | 25 | Sources of funding and other support (such as supply of drugs), role of funders | 39 |
|  | 26 | Ethical approval or approval by research review committee, confirmed with reference number | 6 |

Citation: Eldridge SM, Chan CL, Campbell MJ, Bond CM, Hopewell S, Thabane L, et al. CONSORT 2010 statement: extension to randomised pilot and feasibility trials. BMJ. 2016;355. This is an Open Access article distributed in accordance with the terms of the Creative Commons Attribution (CC BY 3.0) license (<http://creativecommons.org/licenses/by/3.0/>), which permits others to distribute, remix, adapt and build upon this work, for commercial use, provided the original work is properly cited.

**Supplementary Material B: Changes to the trial methods after pilot trial commenced, but before any participants commencing the intervention**

| **Changes to trial methods** | **Initial pilot trial plan as registered with Australian New Zealand Clinical Trials Registry (ANZCTR)** | **Changes to pilot trial and reasoning as updated on ANZCTR** |
| --- | --- | --- |
| Reduction of target parent sample size | “A sample size of 30-35 parents will be recruited to receive the intervention.The PiP+ trial suggests that pre-post intervention effect sizes for primary and secondary outcomes (not specific to suicide prevention) were medium (d=0.59 for carer burden) to very large (d=1.44 for parenting self-efficacy). The absence of effect size estimates on the suicide-prevention-specific outcomes precludes a precise calculation of the required sample size. Using SPSS Sample Power, N=27 provides 71% power to detect an effect size d=0.5 and 85% power for d=0.6, with a=.05, and pre-post correlations=.5. We will recruit 30-35 parent-adolescent dyads to allow for up to 30% attrition.” | “A sample size of 15-18 parents will be recruited to receive the intervention. A sample size of 10 has been found to be appropriate for pilot studies estimating sample size (Birkett & Day, 1994) and is in alignment with guidance for evaluating interventions published by the Medical Research Council (Craig et al., 2008). The recruitment of 15-18 parent-adolescent dyads will account for the anticipated lower rates of adolescent participants (i.e. given that parents can participate without their adolescent also participating), and will allow for at least 50% attrition” “We have reduced the sample size of the trial to ensure its feasibility” |
| Removal of quantitative adolescent outcome measure at pre- and post-intervention: Suicidal Ideation Questionnaire-Junior High School Version | “Changes to adolescent suicidality will be assessed via the adolescent self-report Suicidal Ideation Questionnaire-Junior High School Version (SIQ-JR). This is a 15-item self-report questionnaire designed to measure current suicidal ideation. The measure uses a seven-point scale, ranging from “I never had this thought” (0) to “Almost every day” (6), to assess the frequency of suicidal thoughts. Scores range from 0 to 90.” | “The SIQ-JR was removed to ensure the feasibility of the trial and reduce burden to participants.” |
| Removal of adolescent semi-structured interview reporting on the acceptability of perceived parental changes post parent completion of PiP-SP+, and the addition of an open-text question field included in the adolescent post-intervention assessment | “Acceptability of perceived parental changes by adolescents will be assessed qualitatively via semi-structured interviews. Adolescent interview questions will pertain to whether they noticed any parental changes during and post-intervention including perceived emotional support, and whether these changes were deemed acceptable. All responses will be assessed as a composite secondary outcome.  In terms of interview methods, the interview will be semi-structured, one-on-one, and occur via videoconferencing with a member of the research team who was not the coach of the interviewed parent. For adolescents, the interview will be semi-structured, one-on-one, and occur via videoconferencing with a member of the research team who was not the coach of the adolescent's parent.” | “We have reduced the sample size of the trial to ensure its feasibility. Therefore, interviews with adolescents and focus groups with coaches are beyond the scope of the scaled-down project. As we are no longer conducting interviews with adolescents in the trial, we have added an open-text question field for adolescents in their post-intervention survey to assess qualitative acceptability.  The post-intervention survey for adolescents will include an open-text field for adolescents to provide written feedback via the question “Do you have any comments or suggestions about the PiP-SP+ program and any changes you may have noticed in your parent or your relationship with them? We welcome any feedback.” * |
| Removal of coach focus group reporting on the validity of PiP-SP+ post-intervention | “Coach focus group schedules will be based on items included in the Theoretical Framework of Acceptability (TFA) questionnaire (Sekhon et al. 2022) and theories which have informed the PiP-SP+ intervention design (e.g., the supportive accountability model and persuasive system design model; Mohr et al., 2011; Oinas-Kukkonen, H., & Harjumaa, M., 2009; Yap et al., 2017). Focus groups will be conducted with coaches to understand how acceptable the program was to deliver, and how the acceptability of the intervention could be enhanced.” | “We have reduced the sample size of the trial to ensure its feasibility. Therefore, interviews with adolescents and focus groups with coaches are beyond the scope of the scaled-down project.” |

*Note*. * No responses were received by adolescents in the open-text field.

**Supplementary Material C: PiP-SP+ topics, focus of online module, example of accompanying coaching activity, and example parenting goal**

| **Topic Name** | **Focus of Online Module Content** | **Example Coaching Activity** | **Example Parenting Goal** |
| --- | --- | --- | --- |
| **Understanding anxiety and depression** (core)^a^ | Identify symptoms of anxiety and depression in comparison to normative adolescent development; and provide self-help strategies for anxiety and depressive symptoms. | Guided reflection on which anxiety and depressive symptoms that parent’s adolescent experiences. | Encourage your teenager to try out one of the self-help strategies.  Consider how you could help your teen to remember to do these things regularly. For example, setting a calendar reminder or sending them a reminder text on particular days or times. |
| **Connect** (core)^a^ | Strengthen the parent-adolescent relationship by supporting effective communication. | Evaluate the thoughts, feelings, and behaviors from both adolescent and parent perspectives when in a challenging circumstance or disagreement (e.g., not completing school work). | Use identify, validate & understand techniques in conversations with your teenager. |
| **Suicide prevention: Starting the conversation** (core)^a^ | Develops an understanding of suicidal thoughts/behaviors, identify warning signs, how to talk about suicide with their adolescent, and respond in imminent and non-imminent situations. | Role-play with parent and coach where parents practise having a conversation about suicide with their adolescent and reflect upon the role-play experience. | If you are concerned that your teen might have suicidal thoughts and you haven't had a conversation about suicide with them, ask them if they are having suicidal thoughts. |
| **Suicide prevention: Staying connected** (core)^a^ | Equip parents with the tools to continue conversations about suicide with their adolescent, manage long-term suicidal ideation, and promote the well-being of both the family and the parent. | Guided reflection between coach and parent about adolescent’s other siblings. To explore impact on family dynamic when an adolescent is experiencing suicidal thoughts, and explore perceived benefits in discussing adolescent’s mental health with their other children. | Do a fun activity together as a family this week. Try to find something everyone is happy to do, and involve as many family members as possible. |
| **Understanding non-suicidal self-injury** (optional)^b^ | Examines the interplay between non-suicidal self-injury and suicide. Explores potential reasons for self-injury, and how parents can support their adolescent to develop other coping strategies. | Psycho-education on urge-surfing and how it can be used to manage non-suicidal self-injury. Guided reflection with coach on potential triggers of adolescent’s non-suicidal self-injury, and potential coping strategies that parents could encourage adolescent to engage in when experiencing lower and higher intensity urges. | Support your teen to 'ride the wave'.  Talk with your teen about ‘riding the wave’ and what strategies they could use at different stages of the wave. Together, write a list of some helpful coping strategies to try in the next week. |
| **Raising good kids into great adults** (optional)^b^ | Explores how to set and maintain age appropriate expectations and boundaries. | Explore with parents established expectations within their family. To discuss opportunities of how to create other rules including strategies to uphold consequences. | Thank your teenager for their good behaviour. Tell them you appreciate it, and try to do something special for them. |
| **Partners in problem solving** (optional)^b^ | Equips parents with skills to support their adolescent to effectively problem-solve and to support stress management. | Support parents to help their adolescents overcome a challenge they’re currently facing, by applying the six problem-solving steps. | Talk with your teen about a problem they are experiencing and work with them to apply the 6 problem-solving steps. |
| **Calm vs conflict** (optional)^b^ | Supports parents to manage conflicts with their adolescents more effectively while fostering the development of their adolescent’s conflict resolution skills. | Reflect upon parents' communication styles during conflicts—passive, aggressive, and assertive—and practicing how an assertive responses could be communicated during a conflict with their adolescent. | Next time you have an argument with your teen or another family member, take some time to calm down before trying to resolve the disagreement. |
| **Maintaining the gains** (core)^a^ | Psycho-education on relapse prevention and recovery, and how to support adolescents as they recover. | Reflection activity on parenting strategies which have been used during PiP-SP+ and ones that have been the most helpful. Discuss possible warning signs of relapse for adolescent. Reflect upon the PiP-SP+ experience and parenting strengths.. | **N/A**: final coaching session |
| **Nurture roots and inspire wings** (optional)^c^ | Equips parents with strategies to help support their adolescent to develop age-appropriate autonomy, whilst maintaining parent-adolescent connection. | **N/A** | Discuss with your teen an extra-curricular activity that they might be interested in doing.  Be curious and encourage them to think of activities they may enjoy doing. |
| **Good health habits for good mental health** (optional)^c^ | Supporting healthy habits for adolescent, related to sleep routines, eating, exercise, screentime, and substance use | **N/A** | Talk with your teen about new meals they would like to try. Pick an option with a variety of nutritious foods in it - cook it together, and enjoy! |
| **Breaking the anxiety cycle** (optional)^c^ | Explores the relationship between adolescent anxiety, parent accomodation on anxiety, and avoidance. Equips parents with strategies to help adolescents confront and overcome their fears. | **N/A** | Model helpful anxiety management strategies for your teen.  The next time you are confronted with an anxiety-provoking situation, show your teen how you actively use strategies to deal with your own anxieties**.** |

*Note.* ^a^ core topics which are required to be completed as a part of the program, ^b^ optional and recommended online modules which have a corresponding coaching session, ^c^ optional online module offered without a corresponding coaching session

**Supplementary Material D: Sample content from the PiP-SP+ Action Plan, demonstrating how it aligns with online module materials and coaching sessions.**

The content of the PiP-SP+ Action Plan was designed to empower parents, contents include:

- A ‘Suicide Prevention Action Plan’ to help parents assess when the situation is urgent and which services to contact depending on urgency.
- Adolescent’s care team, to support parents to reflect on available professional and personal supports within their adolescent’s life. To encourage parents to consider their unique carer’s role.
- ‘The urge to self-injure: Learning to ride the wave” plan, which outlines potential triggers which may increase adolescent’s urge to self-injure, and list of strategies parents could suggest when the urge is at lower or higher intensity.
- An activity in which parents reflect on adolescent’s key strengths.
- A parent self-care plan to support their own-wellbeing.
- A program reflections page where parents can reflect upon and journal their learnings.

| **PiP-SP+ action plan resource excerpt** | ‘Suicide Prevention Action Plan’ page introduced to parent during the ‘Suicide Prevention: Starting the Conversation’ topic. Coach provides parent with the appropriate mental health triage phone number and supports parent to feel confident with using plan. 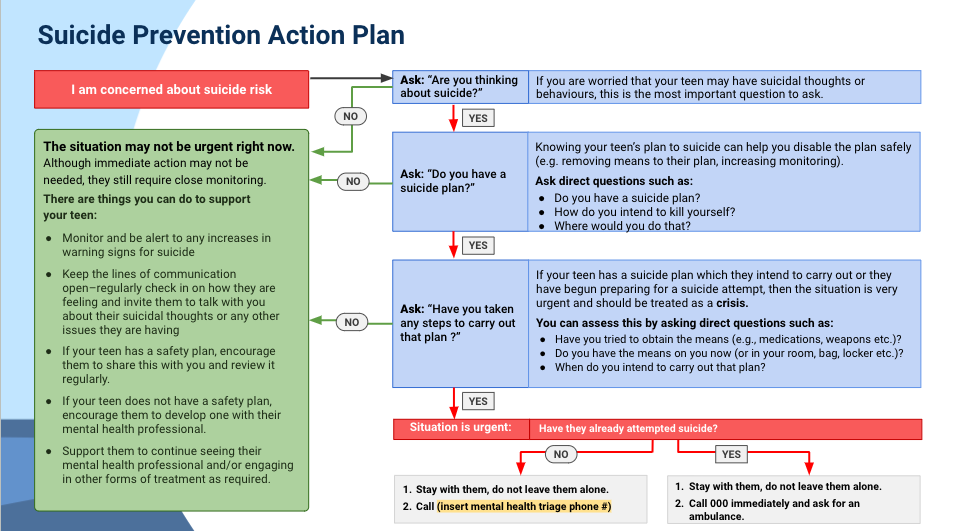 |
| --- | --- |
| **Corresponding online module content** | The content of how to assess for the urgency of the situation is first introduced in the online content. 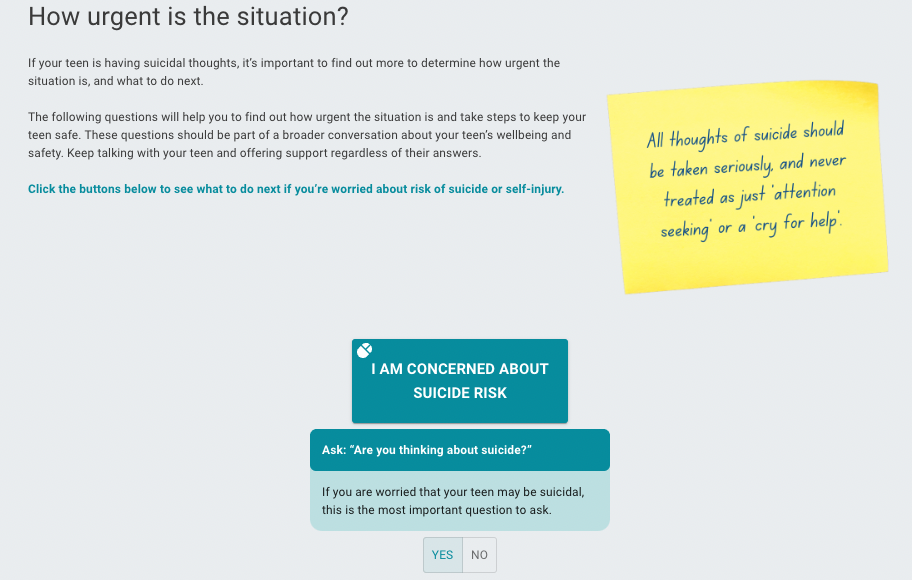 |
| **Corresponding coaching session activity** | During the corresponding coaching session, parents are supported by coaches to practice role-playing how they could approach a conversation about suicide with their adolescent.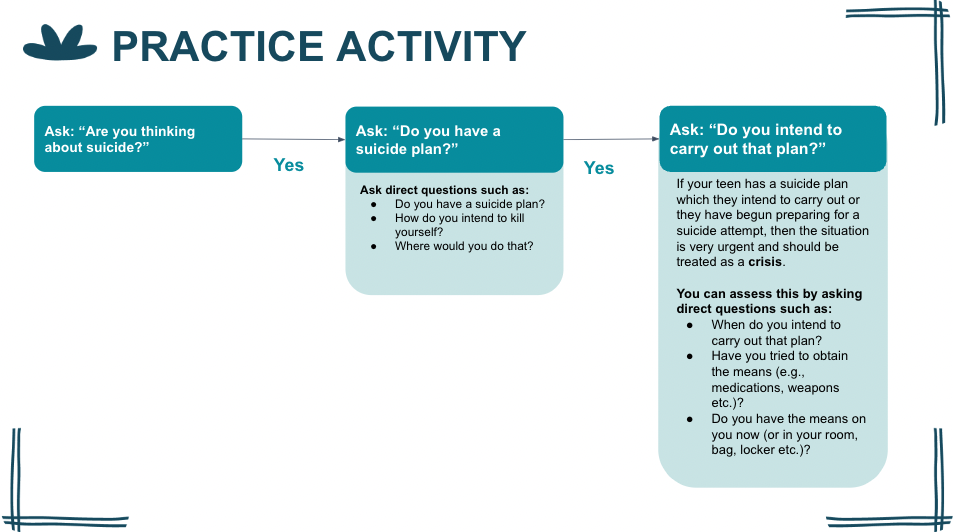 |

**Supplementary Material E: Outcome Measure, Sample Item, and Response Scales**

| Outcome Measure | Sample Item | Response Scale |
| --- | --- | --- |
| Parental self-efficacy to respond to adolescent suicidality | How confident are you that you can ask your child if they are experiencing thoughts of suicide? | 0 (not at all confident) to10 (completely confident), with an anchor of 5 (somewhat confident) |
| Parental self-efficacy to respond to adolescent non-suicidal self-injury | How confident are you that you can ask your child if they are experiencing thoughts of self-injury or has self-injured? | 0 (not at all confident) to10 (completely confident), with an anchor of 5 (somewhat confident) |
| Parenting behaviours protective against adolescent anxiety and depression (PRADAS) | When [my teenager] looks worried, upset, or angry, I show my concern by asking about [his/her] feelings. | Never, rarely, sometimes, often |
| Parental self-efficacy to engage in protective parenting behaviours (PSES) | How confident do you feel about your ability to build and maintain a close relationship with your teenager | Not at all confident (1), a little confident (2), somewhat confident (3), very confident (4) |
| Quality of mental health support intended to be provided (MHSS-I) | How likely is it that you would take the following actions with your teen: Let them know you are listening to what they are saying by restating and summarising what they have said. | Very unlikely (1), Unlikely (2), Neither likely nor unlikely (3), Likely (4), Very likely (5) |
| Carer burden (BAS) | Because of (adolescent name’s) illness, to what extent have you found it difficult to concentrate on your own activities | 1 (Not at all), 2 (A little), 3 (Some), 4 (A lot), 9 (NA) |
| Family functioning (FAD-GF) | There are lots of bad feelings in the family. | Strongly agree (1), Agree (2), Disagree (3), Strongly Disagree (4) |
| Parent psychological distress (K6) | During the past 30 days, about how often did you feel so depressed that nothing could cheer you up? | All of the time (1), Most of the time (2), Some of the time (3), A little of the time (4), None of the time (5) |
| Parent-report of their adolescent’s anxiety symptoms (RCADS-25-P anxiety subscale) | My child worries that something awful will happen to someone in the family | Never (0), Sometimes (1), Often (2), Always (3) |
| Parent-report of their adolescent’s depressive symptoms (RCADS-25-P depressive subscale) | My child feels sad or empty | Never (0), Sometimes (1), Often (2), Always (3) |
| Client Satisfaction Questionnaire (CSQ-8) | If a friend were in need of similar help, would you recommend our service to him or her? | Varied response scale dependent on the item. |
| Perceived parental emotional support if the adolescent was experiencing suicidal ideation or self-injuring | If I had thoughts of killing myself, my parents would provide the emotional support I need | Strongly disagree (0), Disagree (1), Neither agree nor disagree (2), Agree (3), Strongly agree (4) |
| Adolescent self-reported anxiety symptoms (RCADS-25 anxiety subscale) | I worry about things | Never (0), Sometimes (1), Often (2), Always (3) |
| Adolescent self-reported depressive symptoms (RCADS-25 depressive subscale) | I feel sad or empty | Never (0), Sometimes (1), Often (2), Always (3) |

**Supplementary Material F: Semi-structured interview schedule based on items from the Theoretical Framework of Acceptability (Sekhon et al., 2022)**

| **Topic** | **Central Questions** | **Probes/Prompts** |
| --- | --- | --- |
| TFA construct: Affective attitude  “Did you like or dislike [intervention]”  OR  “How comfortable did you feel [to engage with] [intervention]” | **“To start with, it would be great to hear about your overall impressions of the program.”**  **1. Overall, what did you think of the PiP-SP program?**  **2. Overall, did you like or dislike the PiP-SP program?** | **Specific prompts:**  - [If not already covered] What, if anything, did you **like** about PiP-SP?  - [If not already covered] What, if anything, did you **dislike** about PiP-SP?  - How could PiP-SP be **adapted to overcome/improve** [X]?  - Did you experience **discomfort** of any kind as a result of engaging with PiP-SP?  **General probes:**  -Can you tell me more about that?  -How so?  -Why / why is that / why was that the case for you?  -Why is that important to you?  -Could you give me an example? |
| TFA construct: Intervention coherence “The extent to which the participant understands the intervention and how it works (i.e. the ‘face validity’ of the intervention for the recipient)” | - How **similar** was the PiP-SP program to what you **expected**? | **General probes:**  -Can you tell me more about that?  -How so?  -Why / why is that / why was that the case for you? |
| TFA construct: Perceived effectiveness  “The [intervention] has improved [behaviour/condition/clinical outcome]” | **The program was designed to increase parents’ confidence in responding to their teen’s suicidal thoughts, behaviours, and self-injury.**  **3. How helpful was the program, if at all, in building your confidence to respond to [adolescent]’s suicidal thoughts or behaviours?**  **4. How helpful was the program, if at all, in building your confidence to respond to [adolescent]’s non-suicidal self-injury?** *(skip if parent did not complete non-suicidal self-injury module)*  **The program was also designed to develop parent’s skills and knowledge to respond to their teen’s suicidal thoughts/behaviours and self-injury.**  **5. How helpful was the program, if at all, in developing your skills and knowledge to respond to [adolescent]’s suicidal thoughts/behaviours?**  **The program was also designed to help parents recognise their own needs and understand their unique role as a parent in [adolescent]’s care team.**  **6. How helpful was the program, if at all, in supporting you to recognise your needs and understand your unique role as a parent in [adolescent]’s care team?**  **The program was also designed to help parents hold hope that their adolescent’s suicidal thoughts and behaviours can be overcome.**  **7. How helpful was the program, if at all, in supporting you to do this?** | **Specific prompts:**  - [If not already covered] Are there **situations that you feel more confident** in handling since doing the program? [If yes] Could you tell me about one of those situations?  - [For all] What **aspects of PiP-SP contributed to your confidence** increasing in this/these way/s, and how?  [Show ‘components’ slide]: *“What, if anything, from this list contributed to your increase in confidence [besides what you already mentioned]?”*  **-** What **would you have needed from PiP-SP for your confidence** in responding to your teen’s **suicidal thoughts/behaviours** to increase [further]? This could be changes to existing aspects of PiP-SP (as per the list), or any other features/components you think would be helpful to add  **-** What **would you have needed from PiP-SP for your confidence** in responding to your teen’s **self-injury** to increase [further]? This could be changes to existing aspects of PiP-SP (as per the list), or any other features/components you think would be helpful to add  **General probes:**  -Can you tell me more about that?  -How so?  -Why / why is that / why was that the case for you?  -Why is that important to you?  -Could you give me an example? |
| **TFA construct: Ethicality**. The extent to which the intervention has good fit with an individual’s value system.  “How fair is [intervention] for [recipients] with [condition]?”  “There are moral or ethical consequences to engage with [intervention]” | **8. How appropriate was the program to your family context?**  **For example, who is in your family, your cultural or religious background, your financial position, your values towards parenting, education and mental health.** | **Specific prompts:**  -How well did you feel your family context was represented in the program?  - Did you feel excluded or offended by any aspects of the program?  **General probes**  -Can you tell me more about that?  -How so?  -Why / why is that / why was that the case for you?  -Why is that important to you?  -Could you give me an example? |
| **TFA construct: Burden.** The amount of effort required to participate in the intervention.  “How much effort did it take [to engage in] [intervention]? | **9. How much effort did it take for you to do the PiP-SP program?** | **Specific prompts:**  -How did you feel about the amount of effort required of you to do the PiP-SP program?  **General probes**  -Can you tell me more about that?  -How so?  -Why / why is that / why was that the case for you?  -Why is that important to you?  -Could you give me an example? |
| **TFA construct: Opportunity costs.** The benefits, profits or values that were given up to engage in the intervention.  “[Engaging with] [intervention] interfered with my other priorities” | **10. What, if anything, did you need to give up or sacrifice in order to do the PiP-SP program?** | **Specific prompts:**  -How did you feel about having to give up/sacrifice those things in order to do the program?  **General probes**  -Can you tell me more about that?  -How so?  -Why / why is that / why was that the case for you?  -Why is that important to you?  -Could you give me an example? |
| **TFA construct: Confidence in completing program requirements.** That participants’ confidence that they can perform behaviour(s) required to participate in the intervention  “How confident did you feel about [engaging with] [intervention]?” | **11. How confident did you feel about your ability to complete the PiP-SP program?**  **(Requirements)** | **Specific probes:**  How confident did you feel in your ability to…  - do the online modules?  - do the weekly coaching sessions?  - complete your between-session goals?  - make adjustments to your parenting? - use the ‘PiP-SP Action Plan’?  **General probes**  -Can you tell me more about that?  -How so?  -Why / why is that / why was that the case for you?  -Why is that important to you?  -Could you give me an example? |
| **Other suggestions for program improvement** | **12. Do you have any other comments or suggestions for improving the program that we haven’t discussed so far?** | **General probes**  -Can you tell me more about that?  -How so?  -Why / why is that / why was that the case for you?  -Why is that important to you?  -Could you give me an example? |

**Supplementary Material G: Adapted parental self-efficacy to respond to adolescent suicidal crises scale (Czyz et al., 2018)**

For the purpose of this questionnaire, suicidal thoughts and behaviours refers to when a person thinks about or takes actions to intentionally kill themselves.

How confident are you that you can…

1. Ask your child about their mood?
2. Offer emotional support to your child (such as listen to your child, tell your child they are important to you, give them a hug?
3. Assist your child to access treatment services for their difficulties
4. Encourage your child to cope with their difficulties in ways that have been helpful in the past?
5. Ask your child if they are experiencing thoughts of suicide?
6. Respond in a helpful manner if your child discloses thoughts of suicide?
7. Identify suicide indicators or warning signs in your child?
8. Work with your child on a plan for their safety?
9. If your child has thoughts of killing themselves in the future, how confident are you that they will tell you?
10. If your child has thoughts of killing themselves in the future, how confident are you that you will be able to keep your child safe?

**Response scale:** 0 (not at all confident) to10 (completely confident), with an anchor of 5 (somewhat confident)

**Supplementary Material H: Parental self-efficacy to respond to adolescent non-suicidal self-injury scale**

For the purpose of this questionnaire, non-suicidal self-injury refers to where a person deliberately wants to harm themselves (e.g., cutting, burning, or biting) without the intention to end their life.

How confident are you that you can…

1. Ask your child if they are experiencing thoughts of self-injury or has self-injured
2. Respond in a helpful manner if your child discloses self-injury
3. Identify indicators or signs of self-injury in your child?
4. If your child has thoughts of injuring themselves in the future, how confident are you that they will tell you?
5. If your child has thoughts of injuring themselves in the future, how confident are you that you can reduce their risk of self-injury?

**Response scale:** 0 (not at all confident) to10 (completely confident), with an anchor of 5 (somewhat confident)

**Supplementary Material I: Adapted Mental Health Support Scale - Intended (Morgan et al., 2023)**

For the purposes of this project, a ‘mental health problem’ occurs when a person feels depressed, anxious or emotionally stressed over a period of weeks or more, and this interferes with their life. It could include, for example, depression, anxiety disorders, eating disorders, substance use disorders, schizophrenia, bipolar disorder or personality disorders.

Take a moment to think about [adolescent name]. Imagine your [adolescent name] is experiencing a mental health problem, experiencing a worsening of an existing mental health problem, or is in a mental health crisis (e.g. they are suicidal).

How likely is it that you would take the following actions with your teen ?

| 1 | Ask if they have been having thoughts of harming themselves or others. (A) |
| --- | --- |
| 2 | Discuss with them their wishes about privacy and confidentiality. (A) |
| 3* | Listen to their problems and try to provide solutions. (L) |
| 4 | Let them know you are listening to what they are saying by restating and summarising what they have said. (L) |
| 5 | Communicate clearly and simply, and repeat things where necessary. (L) |
| 6* | Tell them they have to get their act together. (G) |
| 7 | Convey a message of hope by telling them help is available and things can get better. (G) |
| 8* | Try to cheer them up by telling them that things don't seem that bad. (G) |
| 9 | Offer them information and resources appropriate to their situation. (G) |
| 10 | Discuss their options for seeking professional help. (EP) |
| 11 | Ask whether they have other supportive people they can rely on. (EO) |
| 12 | Discuss with them whether they are interested in self-help strategies. (EO) |
| For the next few questions, imagine you suspect that this person may be thinking about suicide. How likely is it that you would take the following actions with the person? | |
| 13 | Ask if they have been thinking about suicide. (S) |
| 14* | Tell them how much it will hurt their family and friends if they were to kill themselves. (S) |
| 15* | Try to make them understand that suicide is wrong. (S) |
| 16 | Ask if they have a plan for suicide – for example, how, when and where they intend to die. (S) |
| Imagine this person is at immediate risk of suicide. How likely is it that you would take the following actions? | |
| 17 | Encourage them to get appropriate professional help as soon as possible – for example, see a mental health professional or someone at a mental health service. (I) |
| 18 | Make sure they are not left on their own. (I) |

Note. * denotes item on the Not Recommended Scale and is reverse scored. A: Approach, assess and assist with any crisis. L: Listen and communicate non-judgmentally. G: Give support and information. EP: Encourage the person to get appropriate professional help. EO: Encourage other supports. S: Suicidal behaviour. I: Immediate risk of suicide. P

**Response scale:** Very unlikely (1), Unlikely (2), Neither likely nor unlikely (3), Likely (4), Very likely (5)

**Supplementary Material J: Perceived parental emotional support if suicidal or self-injuring scale**

1. If I had thoughts of killing myself, I would feel comfortable talking to my parent about it
2. If I had thoughts of hurting myself on purpose, I would feel comfortable talking to my parent about it
3. If I had thoughts of killing myself, my parents would provide the emotional support I need
4. If I had thoughts of hurting myself on purpose, my parents would provide the emotional support I need

**Response scale**: Strongly disagree (0), Disagree (1), Neither agree nor disagree (2), Agree (3), Strongly agree (4)
